# Boosting Zn‐Ion Storage Behavior of Pre‐Intercalated MXene with Black Phosphorus toward Self‐Powered Systems

**DOI:** 10.1002/advs.202408549

**Published:** 2024-08-29

**Authors:** Cuiqin Fang, Jing Han, Qingjun Yang, Zhenguo Gao, Di Tan, Tiandi Chen, Bingang Xu

**Affiliations:** ^1^ Nanotechnology Center, School of Fashion and Textiles The Hong Kong Polytechnic University Hong Kong 999077 China

**Keywords:** black phosphorus assembly, fabric triboelectric nanogenerator, molecular engineering, self‐powered system, wearable MXene‐based Zn‐ion capacitor

## Abstract

MXene‐based Zn‐ion capacitors (ZICs) with adsorption‐type and battery‐type electrodes demonstrate high energy storage and anti‐self‐discharge capabilities, potentially being paired with triboelectric nanogenerators (TENGs) to construct self‐powered systems. Nevertheless, inadequate interlayer spacing, deficient active sites, and compact self‐restacking of MXene flakes pose hurdles for MXene‐based ZICs, limiting their applications. Herein, black phosphorus (BP)‐Zn‐MXene hybrid is formulated for MXene‐based ZIC via a two‐step molecular engineering strategy of Zn‐ion pre‐intercalation and BP nanosheet assembly. Zn ions as intercalators induce cross‐linking of MXene flakes with expandable interlayer spacing to serve as scaffolds for BP nanosheets, thereby providing sufficient accessible active sites and efficient migration routes for enhanced Zn‐ion storage. The density functional theory calculations affirm that zinc adsorption and diffusion kinetics are significantly improved in the hybrid. A wearable ZIC with the hybrid delivers a competitive areal energy of 426.3 µWh cm^−2^ and ultra‐low self‐discharge rate of 7.0 mV h^−1^, achieving remarkable electrochemical matching with TENGs in terms of low energy loss, matched capacity, and fast Zn‐ion storage. The resultant self‐powered system efficiently collects and stores energy from human motion to power microelectronics. This work advances the Zn‐ion storage of MXene‐based ZICs and their synergy with TENG in self‐powered systems.

## Graphical abstract

This work proposes a two‐step molecular engineering strategy to tackle the inadequate interlayer spacing, deficient active sites, and compact self‐restacking of MXenes, boosting MXene‐based Zn‐ion capacitor (ZIC) energy storage behavior. By well matching the ZIC with triboelectric nanogenerator, a synergistic self‐powered system can be effectively self‐charged by human motion and then power microelectronics.

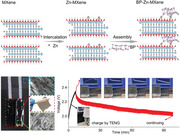

## 1 Introduction

Self‐powered systems are envisioned as promising solutions to the growing demand for lightweight and portable power sources for wearable electronic devices.^[^ ^1^, ^2^ ^]^ A typical self‐powered system integrates energy harvesting, converting, and storing components into a single substrate. On the one hand, substantial advancements have been achieved in harvesting and converting various forms of energy sources into electrical power, such as light, heat, humidity, and mechanical motion.^[^ ^3^, ^4^ ^]^ Among these, triboelectric nanogenerators (TENGs) that can collect mechanical energy from human motion and transform it into microampere‐level electricity have received much attention. On the other hand, metal‐ion batteries, supercapacitors, and Zn‐ion capacitors (ZICs) are representative energy storage devices.^[^ ^5^, ^6^, ^7^, ^8^, ^9^ ^]^ Generally, charging metal‐ion batteries with sluggish electrochemical redox kinetics at intermittent and microampere TENGs is a highly challenging task. Supercapacitors are known for their rapid charging ability, which is achieved by storing energy through ion adsorption.^[^ ^10^ ^]^ However, this energy storage behavior inevitably leads to high self‐discharge rates, posing energy loss challenges for effectively storing the output power of TENGs. Compared with supercapacitors, ZICs incorporating adsorption‐type and battery‐type electrodes are performing better at lowering self‐discharge.^[^ ^11^, ^12^ ^]^ In addition, ZICs with high Coulomb efficiencies (CEs) can be fully charged for short durations.^[^ ^13^, ^14^ ^]^ As a result, the integrated schemes of ZIC and TENG units could be the optimal candidates for self‐powered systems.

Recently, a concept has been devised to integrate ZIC and TENG devices into a self‐powered system, allowing the TENG part to charge the ZIC part to a voltage of 1.8 V at ≈3 µA within 30 min.^[^ ^15^ ^]^ Besides, a synergistic integration of ZIC and TENG units is printed onto a polymer film substrate to form a self‐powered wearable wristband.^[^ ^16^ ^]^ During the self‐charge process, the TENG charges ZIC component at ≈30 µA to support the operation of low‐power electronics. These two cases demonstrate the feasibility of integrating ZIC and TENG units into self‐powered systems. Nevertheless, the self‐discharge behaviors of ZICs as well as the electrochemical matching of ZIC and TENG units have been neglected without further discussion. To our knowledge, the energy storage and anti‐self‐discharge function of ZICs heavily rely on ZIC electrodes, which are crucial for self‐powered systems. MXenes with excellent electrical conductivity and outstanding pseudocapacitive features can appear in various forms, including MXene inks, MXene films, and MXene flakes, which have prominent potential in ZIC electrodes.^[^ ^17^, ^18^ ^]^ Through a divalent cation‐aided gelation strategy, additive‐free MXene ink is proposed to print a cathode for ZIC, delivering a competitive areal capacitance of about 500 mF cm^−2^ and areal energy of 100 µWh cm^−2^ at 0.2 mA cm^−2^.^[^ ^19^ ^]^ Low‐valence Zn atoms are injected into the interlayers of MXene film electrodes for ZICs, reaching an enhanced areal energy of 117 µWh cm^−2^ at 0.5 mA cm^−2^.^[^ ^20^ ^]^ MXene flakes are electrophoretically deposited as electrodes for ZIC, achieving a high areal capacitance of 200.1 mF cm^−2^ and areal energy of 71.6 µWh cm^−2^ at 0.5 mA cm^−2^.^[^ ^21^ ^]^ Unfortunately, the electrochemical performance of these MXene electrodes in ZICs remains limited due to the insufficient interlayer spacing, restrictive active sites, and susceptible self‐restacking of MXenes.

To address the above‐mentioned issues, molecular engineering strategies have been attempted to tailor the surface chemistry and electronic structure of MXenes, including ion pre‐intercalation, molecule pre‐intercalation, and element doping.^[^ ^22^ ^]^ For instance, Sn^2+^ pre‐intercalation expands the interlayer spacing of MXene nanosheets from 1.15 to 1.27 nm; carbon spheres, reduced graphene oxide graphene, and nano‐fibrillated cellulose are inserted into MXene flakes to prevent them from restacking; and additional redox sites are introduced on the surfaces of MXene flake via N‐doping.^[^ ^23^, ^24^, ^25^ ^]^ However, the self‐discharge characteristics of MXene‐based ZICs are often overlooked. Hybridization with other smaller lateral‐size two‐dimensional (2D) materials has been demonstrated to be effective for MXene materials in other types of energy storage devices. For instance, black phosphorus (BP) is an emerging 2D material that has been doped with MXenes for use in supercapacitors, Li‐ion storage, K‐ion capacitors, and Na‐ion storage, showing superior ion energy storage capability.^[^ ^26^, ^27^, ^28^, ^29^ ^]^ Furthermore, BP‐derived phosphorene has been proven to possess exciting anti‐self‐discharge properties in organic solvent ZIC systems.^[^ ^30^ ^]^ However, the Zn‐ion energy storage behavior of BP‐MXene hybrid remains unknown to date.

Herein, we consciously develop the BP‐Zn‐MXene hybrid through a two‐step molecular engineering strategy of Zn‐ion pre‐intercalation and BP nanosheet assembly, aiming to provide upgraded energy storage and anti‐self‐discharge capabilities for MXene‐based ZICs. In the hybrid, MXene flakes with large lateral dimensions are cross‐linked by a trace amount of Zn ions, forming a gel structure with enlarged interlayer spacing. Subsequently, BP nanosheets with small lateral sizes are adorned on the surfaces of the Zn‐MXene gel skeleton. Finally, a wearable ZIC printed with the hybrid and electrodeposited Zn nanosheets demonstrates a competitive capacitance of 2.54 F, superhigh CE of 101.1%, and impressive anti‐self‐discharge performance of 7 mV h^−1^. The wearable ZIC is further paired with fabric TENG to form a self‐powered system via rectifier bridge. By regulating the capacitance of wearable ZIC and the output performance of fabric TENG, a well‐matched self‐powered system is successfully constructed to collect and convert mechanical energy from human movement, providing power for microelectronics. The two‐step molecular engineering strategy offers an effective approach for MXene‐based ZICs, which are further matched with low‐power generators to form self‐powered systems.

## 2 Results and Discussion

**Figure 1:**
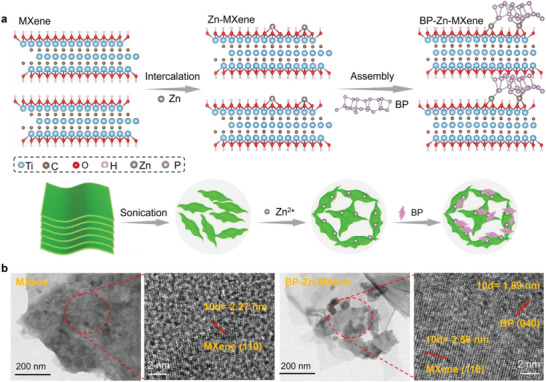
a) Schematic illustration of synthetic process of BP‐Zn‐MXene nanocomposites. b) TEM and HRTEM images of MXene flakes and 10 wt% BP‐Zn‐MXene nanocomposites.

**Figure** **1**a presents the two‐step molecular engineering procedure of MXenes, involving the pre‐intercalation of Zn ions into MXene flakes and the assembly of BP nanosheets onto Zn‐MXene surfaces. Clay‐like multilayered MXenes prepared by etching Ti_3_AlC_2_ with LiF/HCl primarily contain –OH functional groups on their terminal surfaces. After sonication in deionized water, the multilayered MXenes can be well delaminated into flake dispersion. MXene flakes with exceptional surface electronegativity can easily absorb positively charged Zn^2+^ onto their surfaces through electrostatic interactions. A small amount of Zn ions induces the cross‐linking of MXene flakes to form Zn‐MXene gels, enlarges the interlayer spacing, and inhibits the re‐aggregation of MXene flakes. BP nanosheet dispersion (Figure S1, Supporting Information) is obtained by grinding bulk BP, followed by ultrasonic treatment. Owing to the presence of phosphate ions, the BP nanosheet surfaces are negatively charged. Ultimately, the Zn‐MXene gels can spontaneously assemble with BP nanosheets through electrostatic interactions to form BP‐Zn‐MXene nanocomposites.

The scanning electron microscope (SEM) images of as‐prepared samples in each step support the synthetic strategy of BP‐Zn‐MXene nanocomposites (Figure S2, Supporting Information). After assembly, the surfaces of Zn‐MXene gels are covered with BP nanosheets to form BP‐Zn‐MXene nanocomposites. The transmission electron microscope (TEM) and high‐resolution TEM (HRTEM) images of these samples are depicted in Figure 1b and Figure S3 (Supporting Information). The lattice fringe indexed to the (110) plane of MXene flakes expands from 0.227 to 0.258 nm upon the Zn‐ion pre‐intercalation, which promotes rapid and reversible ion migration.^[^ ^26^ ^]^ Following the assembly, BP nanosheets with smaller lateral dimensions are covered on the surfaces of larger MXene flakes. A new lattice distance of roughly 0.189 nm is observed, corresponding to the (040) plane of BP, while the lattice fringe spacing to the (110) plane of MXenes remains almost constant at 0.26 nm.^[^ ^29^ ^]^ As a result, the hybrid provides more active sites, richer ion transport channels, and wider ion diffusion paths for ion adsorption/desorption or insertion/extraction.

**Figure 2:**
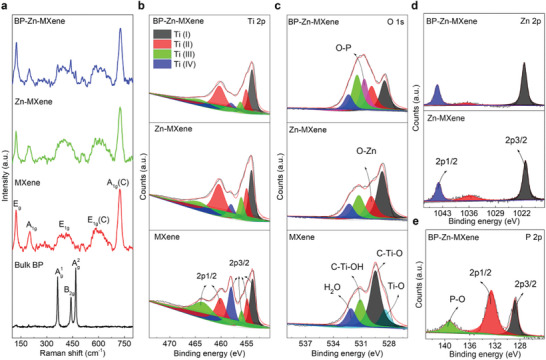
a) Raman spectra of bulk BP, MXene flakes, Zn‐MXene gels, and 10 wt% BP‐Zn‐MXene nanocomposites. b–e) High‐resolution XPS spectra of Ti 2p, O 1s, Zn 2p, and P 2p for MXene flakes, Zn‐MXene gels, and 10 wt% BP‐Zn‐MXene nanocomposites.

**Figure** **2**a discloses the Raman spectra of bulk BP, MXene flakes, Zn‐MXene gels, and BP‐Zn‐MXene nanocomposites. In BP‐Zn‐MXene hybrid, three prominent peaks are identified at ≈363, 439, and 468 cm^−1^, which are attributed to A_g_^1^, B_2g_, and A_g_^2^ of bulk BP, respectively.^[^ ^31^ ^]^ The MXene flakes, Zn‐MXene gels, and BP‐Zn‐MXene nanocomposites share three characteristic peaks at ≈120, 200, and 723 cm^−1^. Additionally, these materials exhibit two broad peak ranges centered around 400 and 600 cm^−1^. These peaks correspond to the E_g_ (Ti, C, T_x_), A_1g_ (Ti, C, T_x_), A_1g_ (C), E_1g_ (Ti, C, T_x_), and E_1g_ (C) vibrations of MXenes, respectively.^[^ ^32^ ^]^ The Raman analysis indicates that BP nanosheets are successfully incorporated into BP‐Zn‐MXene nanocomposites.

To further confirm the surface elemental compositions and chemical states of these samples, X‐ray photoelectron spectroscopy (XPS) spectra are illustrated in Figure S4 (Supporting Information) and Figure 2b–e. The Zn content in Zn‐MXene gels or 10 wt% BP‐Zn‐MXene hybrid is close to 1.7 at% (3.8 wt%), whereas the P content in 10 wt% BP‐Zn‐MXene hybrid is about 9.7 at% (10.6 wt%). The Ti 2p devolution in the range of 450–475 eV comprises four distinct valences, namely Ti (I), Ti (II), Ti (III), and Ti (IV).^[^ ^28^ ^]^ After Zn‐ion pre‐intercalation and BP nanosheet assembly, the proportion of Ti (IV) associated with oxidized Ti weakens, indicating the replacement of oxygen‐containing functional groups. In the O 1s spectra, Zn‐MXene gels and BP‐Zn‐MXene nanocomposites show two additional peaks at ≈529.8 and 530.6 eV, belonging to O‐Zn and O‐P, respectively. According to the Zn 2p spectra of Zn‐MXene gels and BP‐Zn‐MXene hybrid, two main peaks located at ≈1021.2 and 1044.3 eV correspond to Zn 2p_3/2_ and Zn 2p_1/2_.^[^ ^19^ ^]^ Also, the P 2p spectrum of BP‐Zn‐MXene hybrid has three peaks at around 128.7, 132.5, and 139.2 eV, corresponding to P 2p_3/2_, P 2p_1/2_, and P─O bonds.^[^ ^26^ ^]^ Actually, the introduction of P─O bonds is attributed to the oxidation of BP, which improves the electrostatic attraction between BP nanosheets and Zn‐MXene gels. These findings further reveal that Zn and P are incorporated into BP‐Zn‐MXene nanocomposites, forming new surface chemical interactions with the substrate MXene flakes.

**Figure 3:**
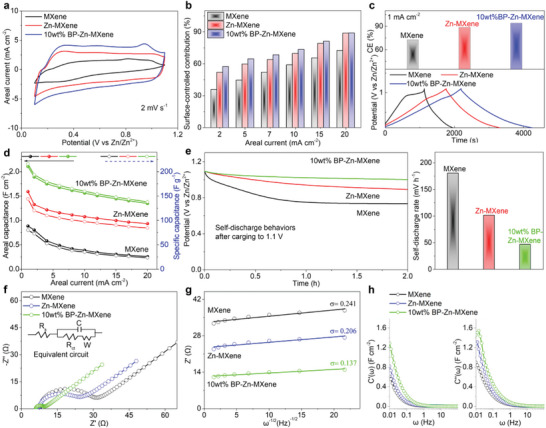
Electrochemical behaviors of MXene, Zn‐MXene and 10 wt% BP‐Zn‐MXene electrodes in 2 m ZnSO_4_. a) CV curves at 2 mV s^−1^. b) Surface‐controlled contributions. c) GCD curves and corresponding CEs at 1 mA cm^−2^. d) Areal and specific capacitances. e) Self‐discharge behaviors. f) Nyquist plots. g) Ion diffusion resistances. h) Real and imaginary capacitances.

The electrochemical behaviors of as‐prepared electrodes are evaluated in 2 m ZnSO_4_ using Zn foils as counter electrodes. At a potential window of 0.1–1.1 V, the MXene electrode can show clear electrochemical signals on the cyclic voltammetry (CV) curve without evident oxidation (Figure S5, Supporting Information). Thus, the potential window for these electrodes is set to 0.1–1.1 V. With the pre‐embedding of Zn ions and the assembly of BP nanosheets, the current signals of CV curves gradually get huge enhancements (**Figure** **3**a, Figure S6, Supporting Information). Weak redox peaks are observed on the quasi‐rectangular CV curves at 2 mV s^−1^ but dissipate with increasing scan rate, implying that the electrochemical energy storage behavior is mainly surface‐controlled and supplemented by diffusion control. On the basis of Dun's formula for response current *i*  = *k*_1_ ν + *k*_2_ *v* ^1/2^, the quantitative contributions of surface control (*k*_1_ *v*) and diffusion control (*k*_2_ *v* ^1/2^) at each scan rate are counted in Figure 3b and Figure S7 (Supporting Information). At 2 mV s^−1^, the surface‐controlled contribution of MXene electrode is only 35.7% due to limited active sites and narrow interlayer spacing. After Zn‐ion pre‐intercalation and BP nanosheet assembly, the surface‐controlled contribution of 10 wt% BP‐Zn‐MXene electrode grows to 57.2%, suggesting accelerated electrochemical reaction kinetics. Among the three BP‐Zn‐MXene electrodes, the 10 wt% BP‐Zn‐MXene electrode demonstrates the relatively largest CV closure area and the highest surface‐controlled contribution at low scan rates, meaning that the dosage of BP nanosheets in BP‐Zn‐MXene hybrid is suggested to be ≈10 wt%.

Figure 3c surveys the galvanostatic charge–discharge (GCD) curves and corresponding CEs of electrodes at 1 mA cm^−2^. For MXene electrode, an obvious charging platform is surveyed at ≈0.95 V, but there is no paired discharging platform, indicating poor charging capability with a low CE of 72.7%. Upon Zn‐ion pre‐intercalation and BP nanosheet assembly, the charging plateau on GCD profile of electrode almost disappears, implying enhanced charging capability with a high CE of 92.9%. As the areal current increases, all GCD curves exhibit almost linear triangular shapes (Figure S8, Supporting Information). Even when the areal current increases to 20 mA cm^−2^, the CE of 10 wt% BP‐Zn‐MXene electrode can be as high as 90.8% (Figure S9, Supporting Information), showing a stable charging ability. The areal capacitance of MXene electrode falls from 0.88 F cm^−2^ (80.1 F g^−1^) to 0.26 F cm^−2^ (23.3 F g^−1^) with a poor rate capability of 29.2% as the areal current climbs from 1 to 20 mA cm^−2^ (Figure 3d, Figure S10, Supporting Information). After Zn‐ion pre‐intercalation and BP nanosheet assembly, the BP‐Zn‐MXene electrodes demonstrate notable improvements in areal capacitance, CE, and rate capability. Among the three BP‐Zn‐MXene electrodes, the 10 wt% BP‐Zn‐MXene electrode performs the highest areal capacitance of 2.11 F cm^−2^ (215.8 F g^−1^) at 1 mA cm^−2^ and 1.35 F cm^−2^ (137.9 F g^−1^) at 20 mA cm^−2^ with a maximum rate capability of 63.9%.

To explore the anti‐self‐discharge performance, the self‐discharge curves of these electrodes are recorded by monitoring the decay of open circuit potentials after being fully charged to 1.1 V (Figure 3e, Figure S11, Supporting Information). Consistently, a sharp decline is observed within the initial 1 h of self‐discharge process, followed by a flatter trend in the subsequent 1 h. This signifies that the open‐circuit potential of these electrodes may quickly fall to a relatively steady potential within 2 h after being completely charged. As a result, the self‐discharge rate during the first 2 h of self‐discharge process is an important indicator for assessing the anti‐self‐discharge performance. Following Zn‐ion pre‐intercalation and BP nanosheet assembly, the self‐discharge rate of electrode dramatically diminishes from 180.6 to 46.5 mV h^−1^. Compared with previously reported supercapacitors and even the carbonaceous ZICs in Table S1 (Supporting Information),^[^ ^11^, ^23^, ^30^, ^33^, ^34^, ^35^, ^36^, ^37^, ^38^, ^39^, ^40^, ^41^, ^42^ ^]^ the BP‐Zn‐MXene system proves superior anti‐self‐discharge performance since the two‐step molecular engineering strategy makes ions absorbed or extracted at the BP‐Zn‐MXene cathode more difficult to self‐diffuse than those absorbed at the carbonaceous electrodes. The improved anti‐self‐discharge capability reduces the energy loss and promotes the charge energy storage of ZICs.

Figure 3f and Figure S12a (Supporting Information) present the Nyquist plots of these electrodes from 0.01 to 100 kHz. Evidently, the MXene electrode reflects the maximum charge transfer resistance *R*_ct_ of 14.1 Ω in the medium‐frequency region, proving the slowest electrochemical reaction kinetics. After Zn‐ion pre‐intercalation and BP nanosheet assembly, the 10 wt% BP‐Zn‐MXene electrode demonstrates the minimum *R*_ct_ of 3.2 Ω, verifying the fastest charge transfer. Besides, the internal series resistance *R*_s_ (close to7.0 Ω) in the high‐frequency region does not undergo obvious changes, indicating that the conductivity of system is relatively stable. The slope of linear fit between square root of frequency (*ω* ^−1/2^) and real resistance (*Z* ^’^) in the low‐frequency region is utilized to estimate the Zn‐ion diffusion resistance σ in the electrolyte (Figure 3g, Figure S12b, Supporting Information). Among these electrodes, the 10 wt% BP‐Zn‐MXene electrode with the lowest *σ* of 0.14 Ω s^−1/2^ is most favorable for rapid ion exchanges. More importantly, a typical capacitive mode *C* (ω) = *C*′ (ω) − *jC*′′(ω) is used to study the capacitive variations over a frequency range of 0.01–100 Hz, where *C*′(ω) and *C*′′(ω) are the real capacitance and imaginary capacitance, respectively. The 10 wt% BP‐Zn‐MXene electrode presents the supreme real capacitance over the frequency range, further certifying the superior capacitive nature (Figure 3h, Figure S12c, Supporting Information). In addition, the relaxation time (1/*ω*) of 10 wt% BP‐Zn‐MXene electrode (68 s) is shorter than that of MXene electrode (100 s), indicating lower energy loss and faster ion transportation.

**Figure 4:**
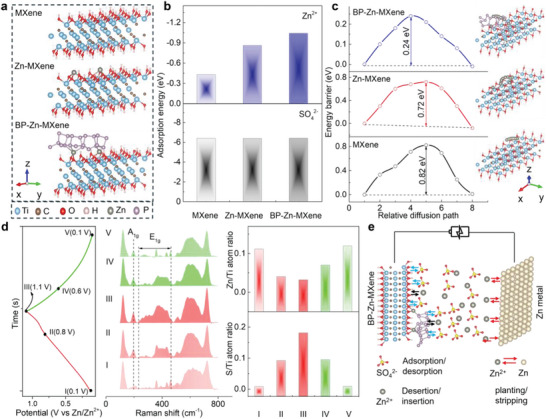
Zn‐ion energy storage mechanism. a) Theoretical models. b) Calculated binding energies of the most stable Zn^2+^ and SO_4_^2−^ adsorption sites. c) Energy barrier and diffusion path of Zn^2+^. d) Raman spectra, Zn/Ti and S/Ti atom ratios of BP‐Zn‐MXene electrode corresponding to five various states during charge–discharge process at 1 mA cm^−2^. e) Schematic illustration of Zn‐ion storage mechanism of BP‐Zn‐MXene electrode.

Theoretical simulations are performed to determine the contribution of Zn‐ion pre‐intercalation and BP nanosheet assembly to the electrochemical performance of MXenes. Density functional theory (DFT) computations are employed to unveil the adsorption and diffusion kinetics of SO_4_^2−^ and Zn^2+^ in MXene, Zn‐MXene, and BP‐Zn‐MXene, respectively. As modeled in **Figure** **4**a, the MXene structure is composed of monolayered Ti_3_C_2_(OH)_2_. The Zn‐MXene structure is achieved by bonding two Zn atoms with O atoms on the monolayered Ti_3_C_2_(OH)_2_ surface, whereas the BP‐Zn‐MXene structure is constructed by positioning a single‐layered BP cluster on the monolayered Zn‐Ti_3_C_2_(OH)_2_ surface. After geometry optimization, the most stable Zn^2+^ and SO_4_^2−^ adsorption sites in these models are pictured in Figure S13 (Supporting Information), and the related adsorption energies can be found in Figure 4b. Impressively, the adsorption energy of SO_4_^2−^ in the structures of MXene, Zn‐MXene, and BP‐Zn‐MXene is almost identical (−6.38 eV), which is more negative than that of Zn^2+^. This indicates that the adsorption of SO_4_^2−^ contributes more to the electrochemical performance than that of Zn^2+^. Comparatively, the adsorption energy of Zn^2+^ in BP‐Zn‐MXene (−1.04 eV) obviously weighs more than that in MXene (−0.43 eV) thanks to more accessible adsorption active sites after the two‐step molecular engineering strategy of Zn‐ion pre‐intercalation and BP nanosheet assembly. Additionally, Figure 4c highlights the diffusion behaviors of Zn^2+^ in the three structures. Along the Zn‐ion diffusion path, the energy barrier of Zn^2+^ diffusion in BP‐Zn‐MXene structure (0.24 eV) is significantly lower than that in MXene structure (0.82 eV). This is attributed to the increased ion migration channels and enlarged ion diffusion paths for Zn ions after the two‐step molecular engineering strategy, confirming the improvement of Zn‐ion diffusion kinetics. The enhanced adsorption and accelerated diffusion capabilities of Zn ions validate that the two‐step molecular engineering strategy of Zn ion pre‐intercalation and BP nanosheet assembly is favorable for storing Zn ions in MXene structure.

To further decipher the Zn‐ion energy storage mechanism, Raman spectra and energy dispersive spectrometer (EDS) are conducted to track the evolution of surface composition of 10 wt% BP‐Zn‐MXene electrode at five marked states during the GCD process at 1 mA cm^−2^ (Figure 4d). Prior to charging, the distinctive peaks (200, 233–463 cm^−1^) denoting the A_1g_ and E_1g_ of surface functional groups of MXene and BP can be detected. As the charging progresses, the intensities of these fingerprint peaks progressively strengthen without any apparent peak position shift. During the discharge process, the peak intensities reverse. These facts may signal the adsorption and desorption of SO_4_^2−^ on the surfaces of BP‐Zn‐MXene hybrid during charge–discharge process. In line with EDS data, the S/Ti atom ratio enhances during charging and reduces during discharging, further conforming to the adsorption/desorption of SO_4_^2−^. However, the alteration in Zn/Ti atom ratio is opposite to that in S/Ti atom ratio. This can be expressed as extracting Zn ions from BP‐Zn‐MXene electrode during charging but inserting them into the electrode during discharging. Furthermore, Figure S14 (Supporting Information) illustrates the surface morphologies of BP‐Zn‐MXene electrode during the charge–discharge process. Before charging, the surface of BP‐Zn‐MXene electrode does not exhibit any nanosheets. Following discharging, the electrode develops a surface architecture with nanosheet growth owing to the Zn^2+^ insertion. After 25 GCD cycles, the Zn/Ti ratio slightly increases with the growth of more nanosheets (Figure S15, Supporting Information). As outlined in Figure 4e, the BP‐Zn‐MXene electrode undergoes adsorption/desorption of negatively charged SO_4_^2−^ and extraction/insertion of positively charged Zn^2+^ during the charge–discharge process.

**Figure 5:**
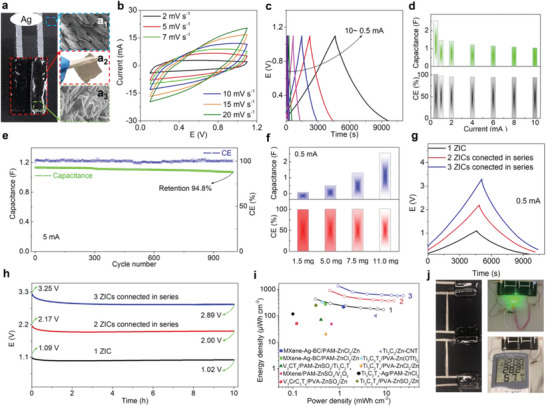
Wearable in‐plane ZICs. a) Construction configuration. a_1_–a_3_) Textile substrate, PAM‐ZnSO_4_ gel and electrodeposited Zn nanosheets. b) CV curves. c) GCD curves at various currents. d) Capacities and CEs at different currents. e) Cycle stability. f) Capacities and CEs at various cathode load masses. g) GCD curves of devices connected in series. h) Self‐discharge curves of devices connected in series. i) Ragone plot comparison with other quasi‐solid‐state MXene‐based ZICs. j) Photographs of powering green LED lights and thermo‐hygrometer.

10 wt% BP‐Zn‐MXene cathode (2 × 0.5 cm^2^) is printed on commercial cloth to exemplify its practical application in a wearable in‐plane ZIC system, where a piece of polyacrylamide (PAM)‐ZnSO_4_ gel and electrodeposited Zn nanosheets are utilized as electrolyte and anode (**Figure** **5**a). All CV curves of the wearable ZIC with a cathode load mass of 11.0 mg maintains quasi‐rectangular shapes without obvious deformation (Figure 5b), well‐documenting excellent capacitive nature. Figure 5c depicts the GCD profiles in the current range of 0.5–10 mA. As the current rises, the charging and discharging durations consistently are almost identical, presenting ultra‐high CEs of 101.1–93.8% (Figure 5d). The wearable ZIC delivers a high capacitance of 2.54 F at 0.5 mA and remains 1.02 F even at a high areal current of 10 mA, suggesting high‐rate capability. After 1000 GCD cycles at 5 mA, the wearable ZIC reaches a capacitance retention of 94.8% and stable CE of up to 98.0%, representing superb cycling stability without obvious influence from Zn dendrites (Figure 5e, Figure S16, Supporting Information). Furthermore, 10 wt% BP‐Zn‐MXene cathodes with various load masses are applied to wearable ZICs. At 10 mV s^−1^, the enclosure area of CV curve increases with the increase of load mass, indicating the variability of capacitive storage (Figure S17, Supporting Information). Based on the GCD curves (Figure S18, Supporting Information), the capacitances and CEs of wearable ZICs with various cathode load masses are calculated in Figure 5f and Figure S19 (Supporting Information). Obviously, all wearable ZICs possess superhigh CEs within the current range, always over 91.3%, suggesting fast charging capability. When the cathode load mass is reduced to 7.5, 5.0, and 1.5 mg, the capacitance of wearable ZIC at 0.5 mA drops to 1.27, 0.48, and 0.07 F, respectively. Besides, the *R*_s_ of wearable ZICs with different cathode load masses is almost equal to 11.2 Ω (Figure S20, Supporting Information), manifesting steady electrical nature.

To broaden the voltage window for powering microelectronic devices, wearable ZICs (cathode load mass of 11.0 mg) are connected in series with Ag paste. The voltage windows of wearable devices with two and three ZICs connected in series are extended to 2.2 and 3.3 V, showing almost identical charging and discharging durations as that of single wearable ZIC at the same current (Figure 5g, Figure S21, Supporting Information). Accordingly, the wearable devices with two and three ZICs connected in series deliver capacitances of 1.34 and 0.90 F at 0.5 mA, respectively (Figure S22, Supporting Information). After being fully charged to 1.1 V, the open circuit voltage (*V*_oc_) of single wearable ZIC only decays by 70 mV within 10 h of idle period, demonstrating an ultra‐low self‐discharge rate of 7.0 mV h^−1^. Even when three ZICs are connected in series, the self‐discharge rate still presents a low value of 36 mV h^−1^, indicating outstanding anti‐self‐discharge performance. This is because Zn is difficult to spontaneously convert into Zn^2+^ at the electrodeposited Zn nanosheet anode under open circuit state, while the ions absorbed or extracted at the BP‐Zn‐MXene cathode can only slowly self‐diffuse into the gel electrolyte. In Figure 5i and Figure S23 (Supporting Information), the wearable ZIC displays the highest areal energy of 426.3 µWh cm^−2^ (42.6 Wh kg^−1^) at the areal power of 0.31 mW cm^−2^ (30.7 W kg^−1^), apparently surpassing other recently reported MXene‐based ZICs (Table S2, Supporting Information).^[^ ^18^, ^19^, ^20^, ^21^, ^43^, ^44^, ^45^, ^46^, ^47^, ^48^ ^]^ This is owing to the fact that larger interlayer spacing, more accessible active sites, and ion transport channels are achieved for MXene flakes after two‐step molecular engineering strategy. The areal energy of wearable device with three ZICs connected in series is up to 1367.4 µWh cm^−2^, successfully powering green LEDs and thermo‐hygrometer (Figure 5j). This showcases the feasibility of wearable ZICs in practical applications.

**Figure 6:**
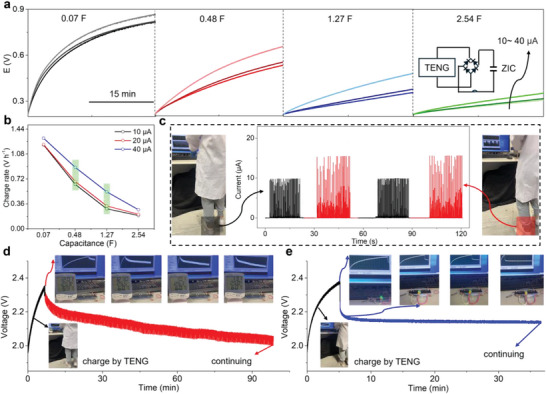
Self‐powered system integrating wearable ZIC and fabric TENG components. a,b) Charging curves and charging rates under different capacities and *I*_sc_ values. c) Output current signals of fabric TENG during human motion. d,e) Illustration of charge–discharge process to power a thermo‐hygrometer and green LED lights.

The synergic principle of fabric TENG and wearable ZIC is proposed to visualize a self‐powered system (Figure S24, Supporting Information), which includes two main units, namely a wearable ZIC device as energy storage component and a fabric TENG device as energy harvesting component. The intermittent alternating current power output from the fabric TENG is converted into direct current power through a bridge rectifier and stored in wearable ZIC component, suggesting the self‐charging process of self‐powered system. The fabric TENG composed of nylon fabric/Cu‐Ni fabric electrode and Ecoflex silicone rubber film/Cu‐Ni fabric electrode operates in contact‐separation mode to achieve electrical energy output. Figure S25 (Supporting Information) collects all output current and voltage signals of fabric TENG under a series of load pressures and frequencies. The generated short circuit current (*I*_sc_) and *V*_oc_ normally fall within the range of 6–40 µA and 100–615 V, respectively. When the frequency is fixed at 4 Hz, the *I*_sc_ can generally reach 10, 20, and 40 µA by regulating the load pressure to 2, 5, and 20 N, respectively. **Figure** **6**a exhibits the charging curves of self‐powered system under different capacitance and *I*_sc_ values. After charging for 30 min at 10 µA, the *V*_oc_ of self‐powered system with a capacity of 0.07 F increases from 0.21 to 0.82 V. As the *I*_sc_ value enhances to 40 µA, the *V*_oc_ reaches 0.87 V after charging for 30 min. As the capacity gradually increases to 2.54 F, the *V*_oc_ can only achieve 0.35 V after charging for 30 min at 40 µA.

Obviously, the charge rate of self‐powered system augments with the increase of *I*_sc_ value but declines with the growth of capacity (Figure 6b). However, the difference in charge rate of the self‐powered system with a capacity of 0.07 F is inconspicuous at various *I*_sc_ values, only 96 mV h^−1^. This is due to the high self‐recovery ability of wearable ZIC component, which can autonomously improve the *V*_oc_ of self‐powered system during rest time (Figure S26, Supporting Information). Moreover, the self‐powered system with a capacity of 2.54 F manifests a small charge rate difference of 89.4 mV h^−1^ under different *I*_sc_ values, indicating that fabric TENG requires a long time to charge high‐capacity wearable ZIC component. When the capacity of self‐powered system is within the range of 0.48–1.27 F, a significant difference of ≈246 mV h^−1^ in charge rate can be achieved, demonstrating the high matchability of wearable ZIC component and fabric TENG in self‐powered system. The fabric TENG can be driven by human feet, harvesting mechanical energy from human motion and converting it into direct current (Figure 6c). The fabric TENG designed for human motion is matched with wearable ZIC component to construct a self‐powered system with a capacity of 0.90 F. After walking for 6.8 min, the voltage of self‐powered system increases from 1.96 to 2.31 V to power a thermo‐hygrometer for over 91.9 min, indicating successful self‐charging through walking (Figure 6d, Movie S1, Supporting Information). Another application of self‐powered device is shown in Figure 6e and Movie S2 (Supporting Information). After walking for 5.2 min, the self‐powered device continuously lights up green LEDs for more than 30 min. Also, the self‐powered system can power an electronic timer for more than 4 h without obvious voltage drop after walking 5 min (Figure S27, Supporting Information). These facts demonstrate the well‐matched synergy of wearable ZIC component and fabric TENG in self‐powered systems.

## 3 Conclusion

In summary, we propose a two‐step molecular engineering strategy to fabricate BP‐Zn‐MXene hybrid using Zn‐ion pre‐intercalation and BP nanosheet assembly for high‐performance wearable MXene‐based ZICs, further coupled with fabric TENG for self‐powered systems. Experimental and theoretical results demonstrate that the hybrid with expandable interlayer spacing enables ample active sites for ion adsorption or insertion, and efficient ion channels for rapid ion transport. Accordingly, the hybrid cathode delivers a much‐improved areal capacitance of 2.11 F cm^−2^ and a strong anti‐self‐discharge capability of 46.5 mV h^−1^. A wearable ZIC with the hybrid cathode holds an impressive capacity of 2.54 F, superhigh Coulombic efficiency of 101.1%, high areal energy of 426.3 µWh cm^−2^, and robust anti‐self‐discharge ability with a self‐discharge rate of 7.0 mV h^−1^. Furthermore, the wearable ZIC component is well matched with fabric TENG to form a self‐powered system that successfully harvests energy from human motion to power microelectronic devices. Hence, the two‐step strategy of Zn‐ion pre‐intercalation and BP nanosheet assembly suggests the potential application of MXene‐based ZICs. This encourages the synergy of wearable ZIC and TENG components in self‐powered systems, achieving integrated energy management of human motion, energy storage, and microelectronic appliances.

## 4 Experimental Section

### Materials

Clay‐like MXene (T_3_C_2_T_x_, mostly T_3_C_2_(OH)_2_) powder and bulk BP were purchased from Xinxi Technology and Hefei Keliao New Materials Technology. Zn foil and Ag paste were purchased from Keloude Technology. ZnSO_4_·7H_2_O, acrylamide (AM), ammonium persulfate (APS), polyvinylidene fluoride (PVDF), activated carbon (AC), acetylene black, and *N*‐methyl‐2‐pyrrolidone (NMP) were purchased from Shanghai Aladdin Bio‐Chem Technology. Ecoflex 00‐50 silicone rubber was purchased from Smooth‐On.

### Preparation of Zn‐MXene Gels and BP Nanosheets

First, 30 mg of clay‐like MXene was sonicated in 20 mL of deaerated water for 30 min to obtain MXene flake dispersion. Subsequently, 2.4 mL of ZnSO_4_·7H_2_O (5 mg mL^−1^) was mixed with the above MXene flake dispersion and stirred for 24 h to achieve Zn‐ion pre‐intercalation and form a uniform Zn‐MXene gel dispersion. Then, 50 mg of bulk BP was first ground in a mortar for 10 min and then sonicated in 100 mL of deaerated water. The whole sonication process was carried out in an ice bath for 8 h to guarantee a temperature below 4 °C. Following sonication, the BP dispersion was separated through centrifugation at 3500 rpm for 15 min, and the supernatant containing BP nanosheets was collected for further use. The concentration of BP nanosheet dispersion (0.15 mg mL^−1^) was determined by measuring the weight change after filtering a known volume of BP nanosheet dispersion on filter membranes.

### Synthesis of BP‐Zn‐MXene Nanocomposites

Typically, the above deaerated Zn‐MXene gel dispersion (22.4 mL) was thoroughly mixed with the above deaerated BP nanosheet dispersion under stirring for 24 h. Then the homogeneous mixture was collected via vacuum filtration. Afterward, the mixture was immediately frozen with liquid nitrogen and then dried under vacuum to obtain BP‐Zn‐MXene powder. For comparison, the dosage of BP nanosheets in BP‐Zn‐MXene nanocomposites was set to 5, 10, and 15 wt% using 11, 22, and 35 mL BP nanosheet dispersions, respectively.

### Construction of In‐Plane Wearable ZIC

Commercial polyethylene terephthalate (PET) cloth was chosen as a wearable substrate. Ag paste was printed on the PET cloth to form in‐plane current collectors. A typical wearable ZIC was constructed using electrodeposited Zn nanosheets as anode, 10 wt% BP‐Zn‐MXene electrode as cathode, and PAM‐ZnSO_4_ gel as electrolyte. First, Zn nanosheets were electrodeposited on AC@Ag current collector in 2 m ZnSO_4_ at 50 mA cm^−2^ for 30 min to form anode using Zn foil as counter electrode. Subsequently, 30 mg of BP‐Zn‐MXene, 3 mg of PVDF, and 3 mg of carbon black were ground in 0.25 mL of NMP until homogenous. The mixed slurry was printed on Ag current collector and then vacuum dried at 50 °C for 12 h to prepare cathode. Finally, 3 g of AM and 11.5 g of ZnSO_4_·7H_2_O were dissolved in 20 mL of deionized water, and then 0.01 g of APS was added to the mixed solution with constant stirring. The homogeneous mixture was poured into a petri dish and cured at 80 °C for 2 h to fabricate PAM‐ZnSO_4_ gel electrolyte.

### Self‐Powered System Integrating Wearable ZIC with Fabric TENG

The fabric TENG was prepared as follows: First, the A component and B component of Ecoflex 00‐50 silicone rubber were mixed at a weight ratio of 1:1 under thoroughly stirring for 10 min, and then the mixed liquid was kept at room temperature for 20 min to remove air bubbles. Subsequently, the mixture was cast on ≈78.5 cm^−2^ of Cu‐Ni fabric and cured in an oven at 50 °C for 2 h to form triboelectric electrode. Besides, commercial nylon fabric was combined with Cu‐Ni fabric to form conductive electrode. Finally, the fabric TENG assembled from triboelectric and conductive electrodes was paired with the above wearable ZIC component through a rectifier bridge and conductive wires to form a self‐powered system.

### Computational Methods

DFT computations were employed to perform the simulations. The Perdew–Burke–Ernzerhof functional was used to calculate exchange and correlation energy within generalized gradient approximation. To include van der Waals forces, D3 correction was added as implemented. All structures were relaxed until the residual forces on the free atoms were smaller than 0.02 eV Å^−1^. The climbing image nudged elastic band method was chosen to identify the minimum energy path and saddle points between the initial and final positions and compute the migration energy barrier. Geometry optimizations were carried out using Monkhorst–Pack k‐point meshes of 2 × 3 × 1. To ensure enough area for Zn atoms to move, the Ti_3_C_2_(OH)_2_ surface was represented using a (3 × 6) supercell.

### Characterizations and Electrochemical Measurements

SEM (Tescan VEGA3) equipped with an EDS was utilized to observe micromorphology. Nanomorphology was performed on a HRTEM (Tecnai G2 F20). XPS (Thermo Fisher Scientific Nexsa) was taken with an aluminum Kα X‐ray source. Raman spectroscopy (Renishaw) was employed with an excitation length of 785 nm (0.5% laser power) and a 50× objective. The output current and voltage signals of fabric TENGs and self‐powered system were recorded on an electrometer (Keithley 6514) under the same environment condition (temperature ≈22 °C, humidity ≈64%). All electrochemical measurements were recorded on an electrochemical workstation (CHI660E, Chenhua).

## Conflict of Interest

The authors declare no conflict of interest.

## Supporting information

Supporting Information

Supplemental Movie 1

Supplemental Movie 2

## Data Availability

The data that support the findings of this study are available from the corresponding author upon reasonable request.
