# Supplementary material for: Boosting Zn‐Ion Storage Behavior of Pre‐Intercalated MXene with Black Phosphorus toward Self‐Powered Systems
Source: Adv Sci (Weinh). 2024 Aug 29;11(40):2408549. doi: 10.1002/advs.202408549 (PMC11515922; doi:10.1002/advs.202408549)
Supplement: Supplementary file 1 — Supporting Information [file ADVS-11-2408549-s001.docx]

**Supporting Information**


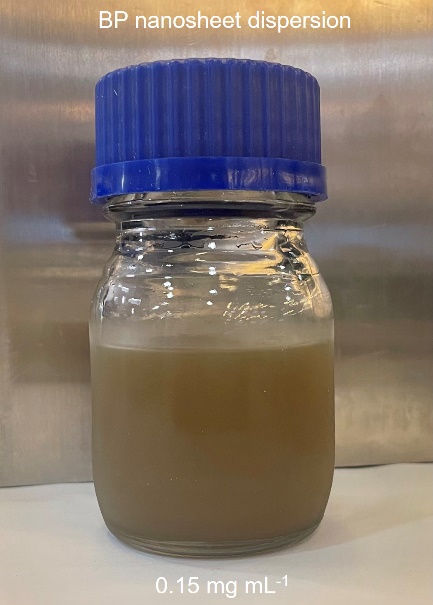


**Figure S1**. Photograph of BP nanosheet dispersion.


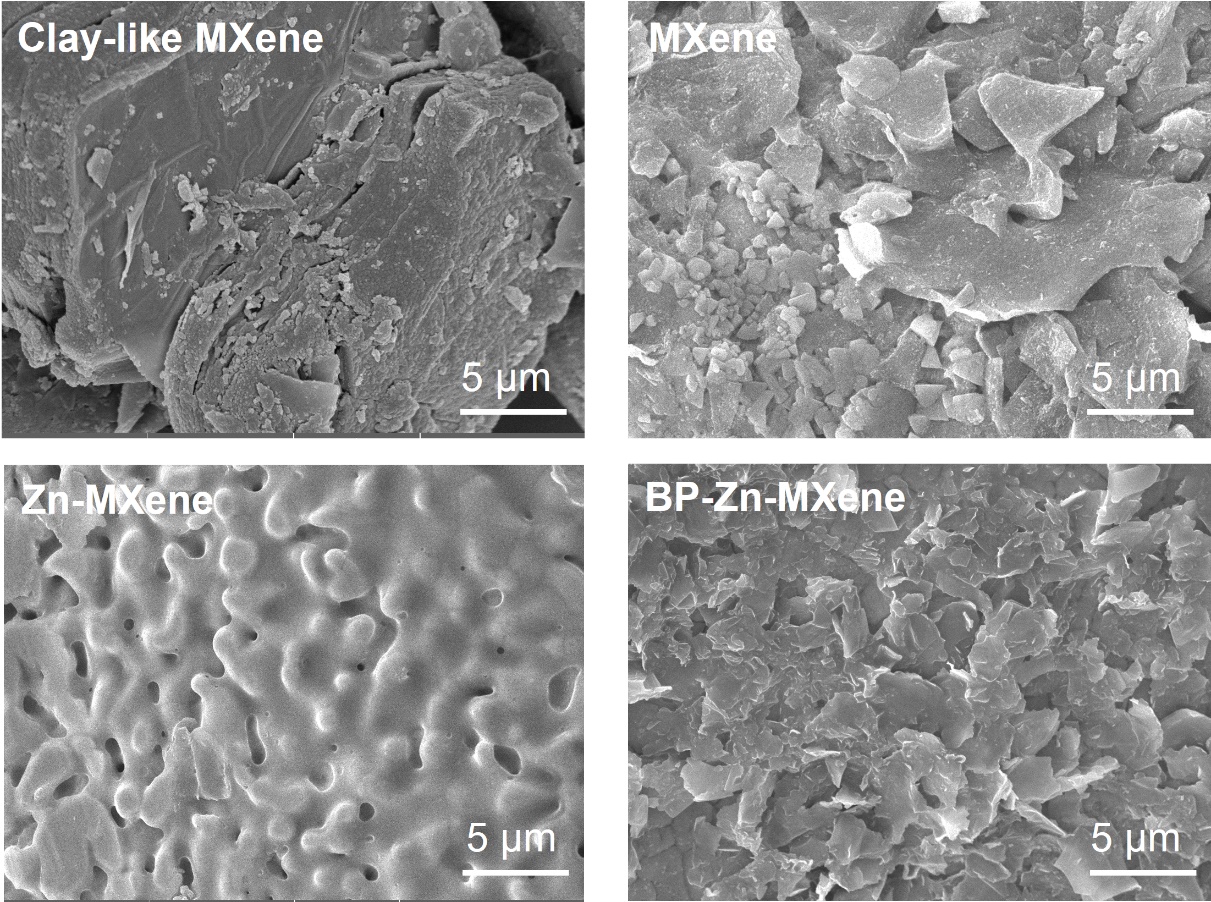


**Figure S2**. Typical SEM images of Clay-like MXene, MXene flakes, Zn-MXene gels and 10wt% BP-Zn-MXene nanocomposites.


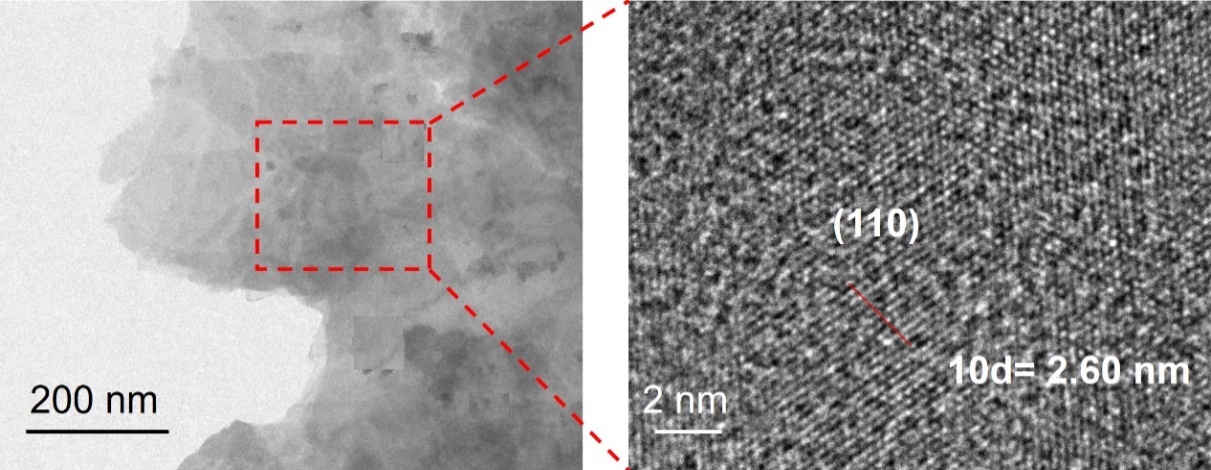


**Figure S3**. TEM and HRTEM images of Zn-MXene gels.


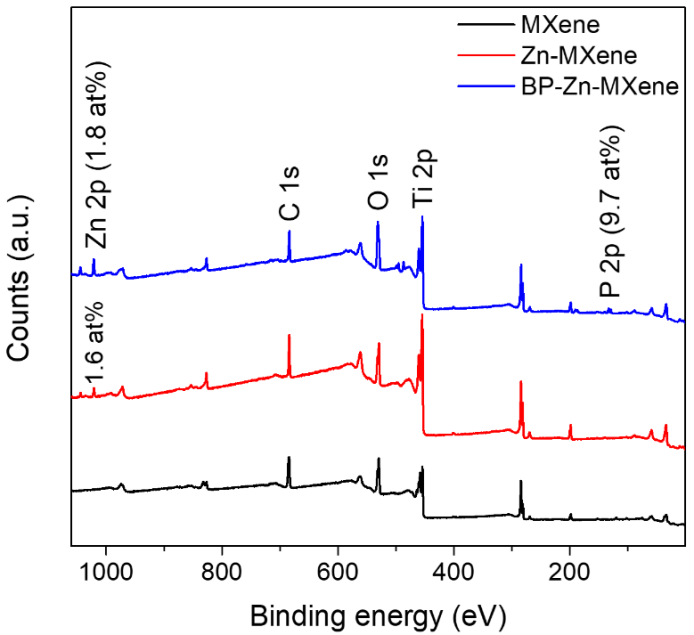


**Figure S4**. Full XPS spectra of MXene flakes, Zn-MXene gels and 10wt% BP-Zn-MXene nanocomposites.


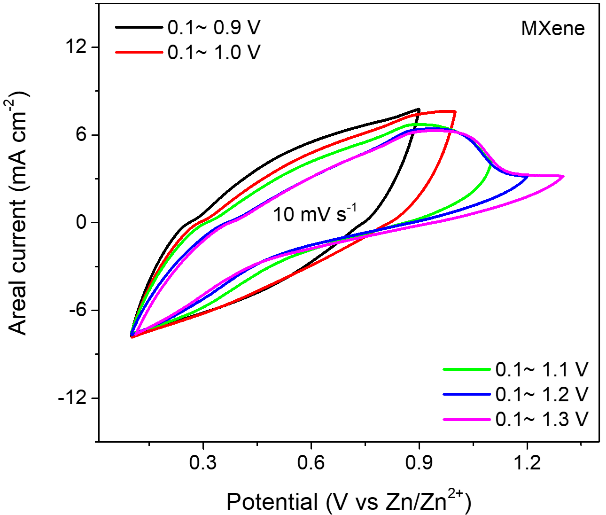


**Figure S5**. CV curves at 10 mV s^-1^ under different potential windows for MXene electrode in 2 M ZnSO_4_.


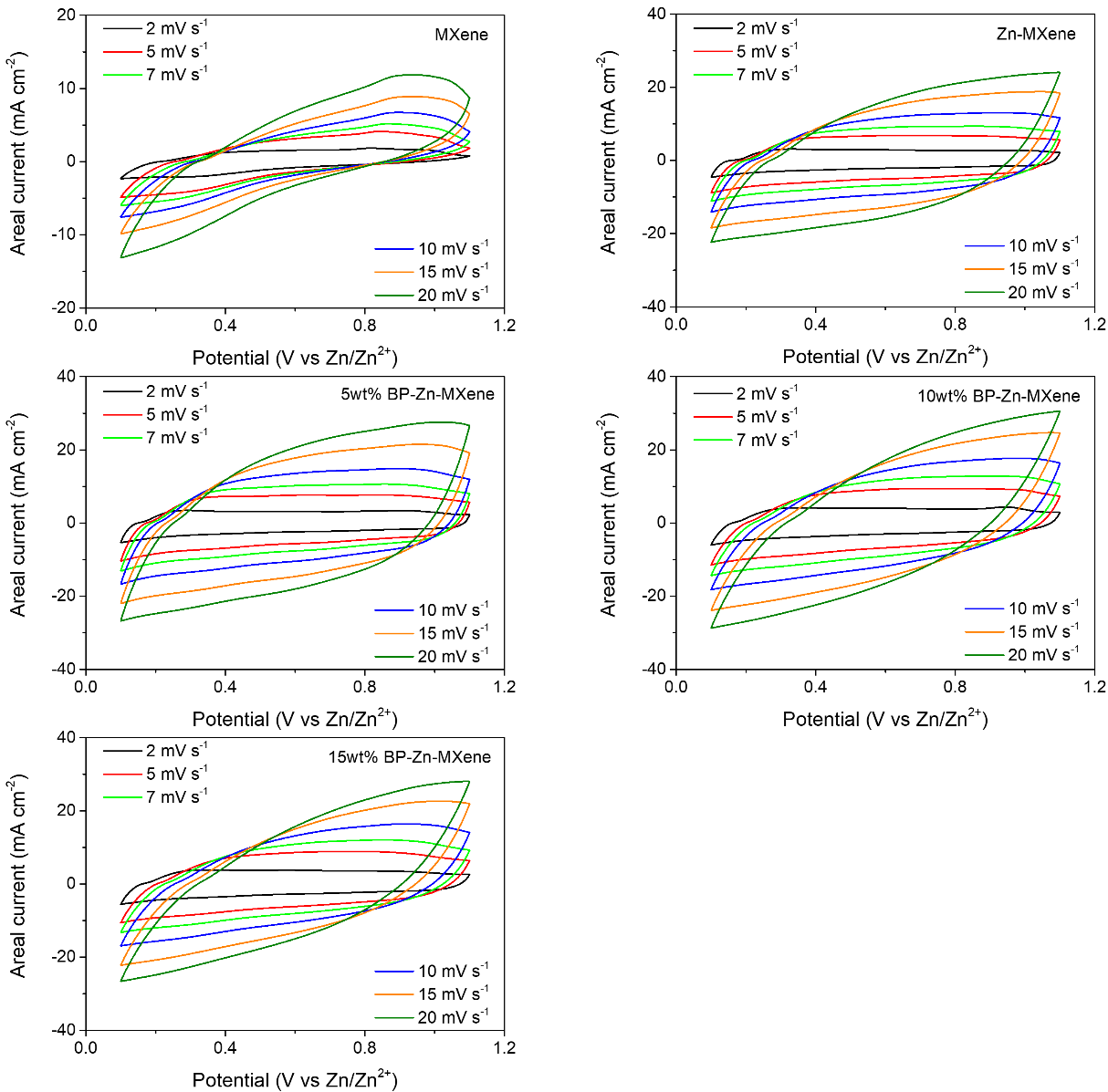


**Figure S6**. CV curves at a series of scan rates for MXene, Zn-MXene, 5wt% BP-Zn-MXene, 10wt% BP-Zn-MXene and 15wt% BP-Zn-MXene electrodes in 2 M ZnSO_4_.


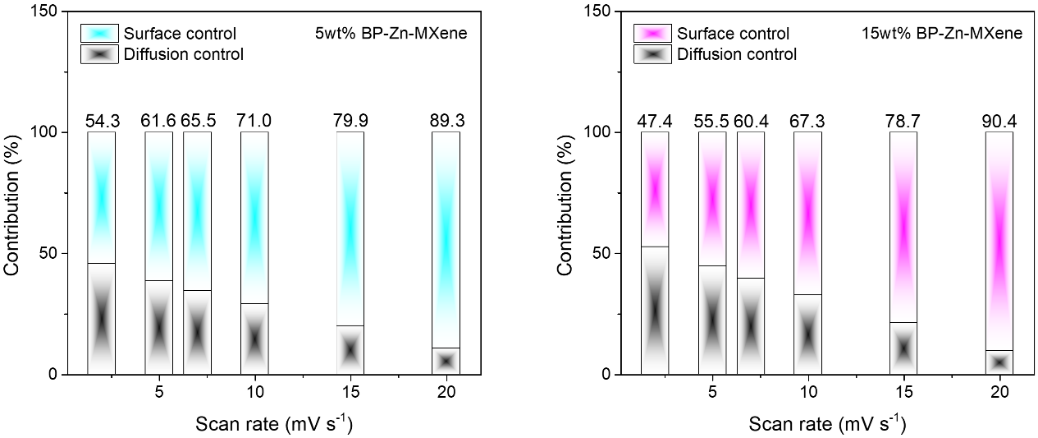


**Figure S7**. Surface-controlled contributions at a series of scan rates for 5wt% BP-Zn-MXene and 15wt% BP-Zn-MXene electrodes in 2 M ZnSO_4_.


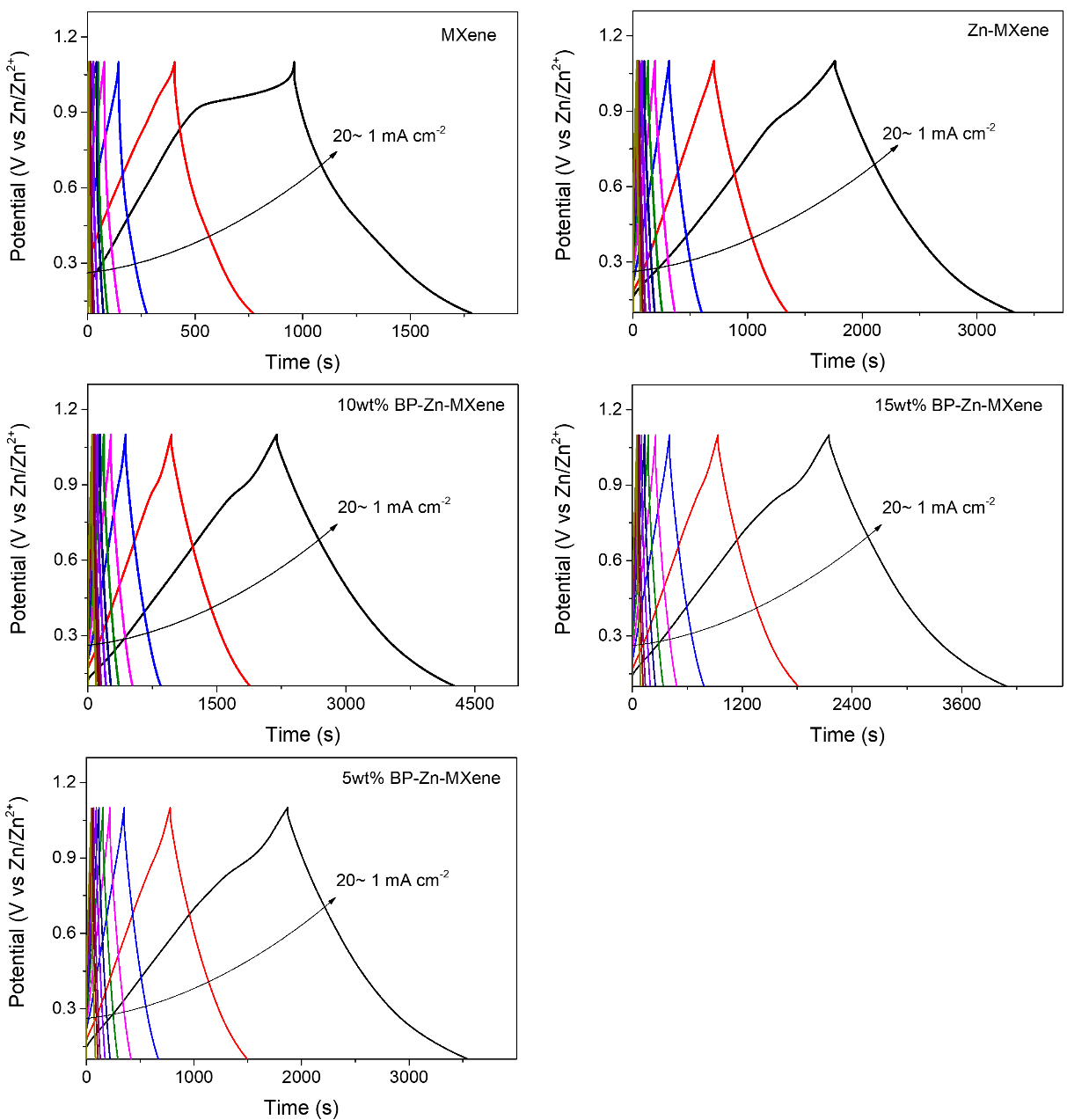


**Figure S8**. GCD curves at a series of areal currents for MXene, Zn-MXene, 5wt% BP-Zn-MXene, 10wt% BP-Zn-MXene and 15wt% BP-Zn-MXene electrodes in 2 M ZnSO_4_.


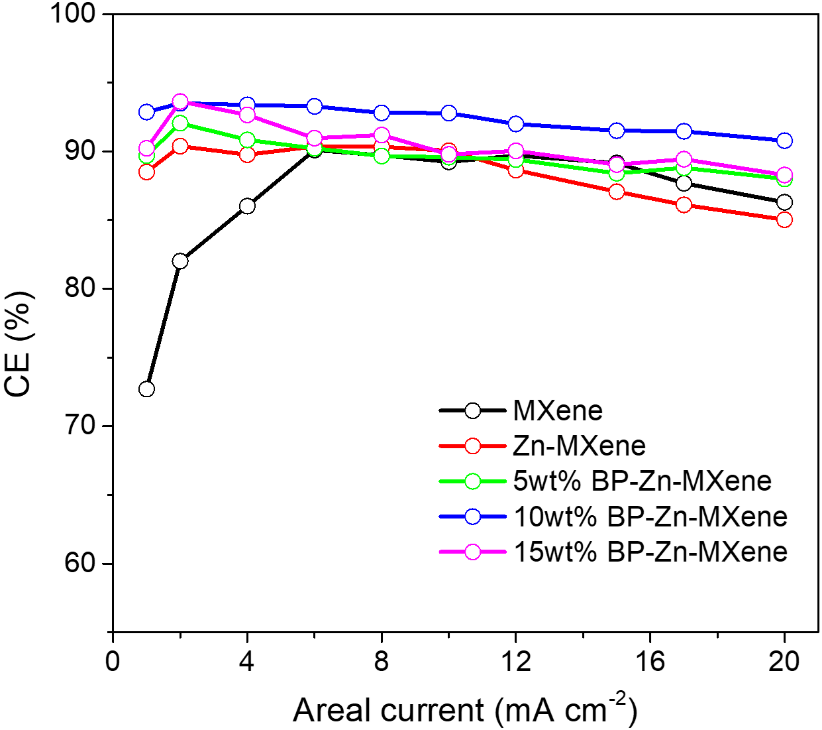


**Figure S9**. Coulomb efficiencies of MXene, ZN-MXene, 5wt% BP-Zn-MXene, 10wt% BP-Zn-MXene and 15 wt% BP-Zn-MXene electrodes at various areal currents in 2 M ZnSO_4_.


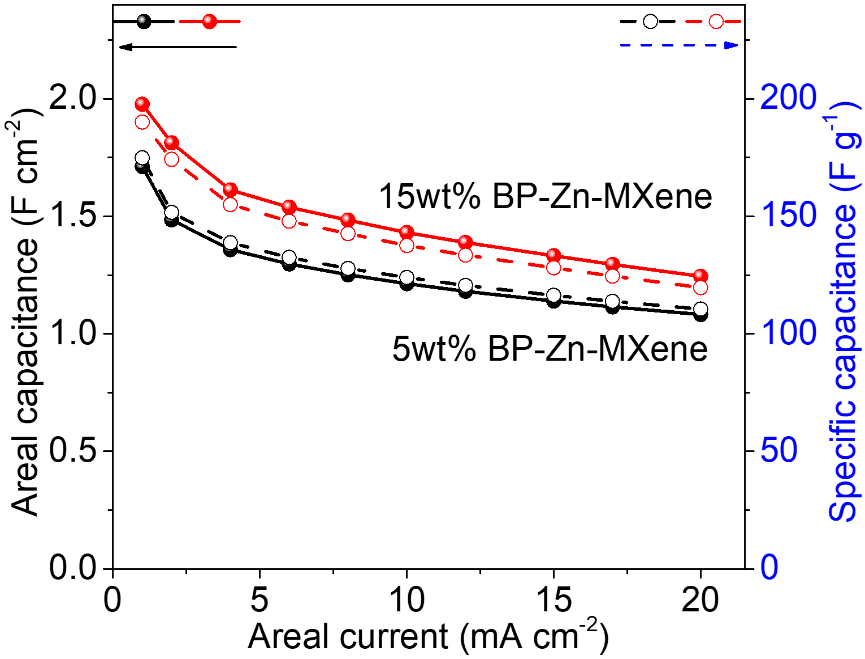


**Figure S10**. Areal and specific capacitances of 5wt% BP-Zn-MXene and 15 wt% BP-Zn-MXene electrodes in 2 M ZnSO_4_.


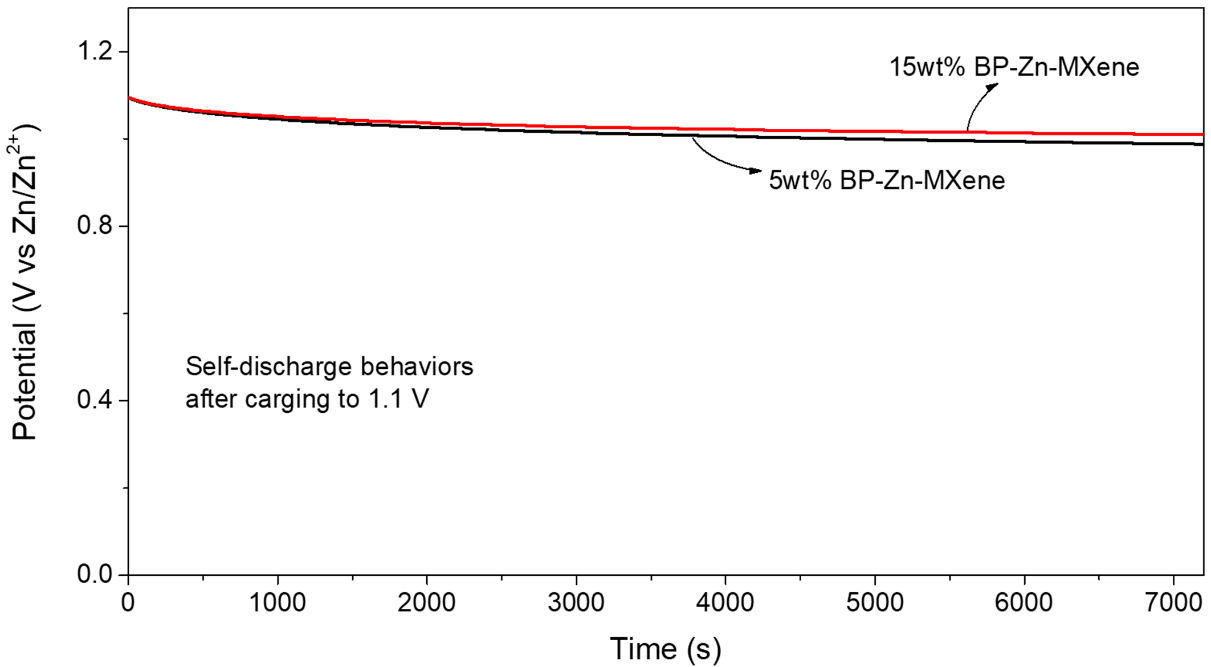


**Figure S11**. Self-discharge curves of 5wt% BP-Zn-MXene and 15 wt% BP-Zn-MXene electrodes after charging to 1.1 V in 2 M ZnSO_4_.


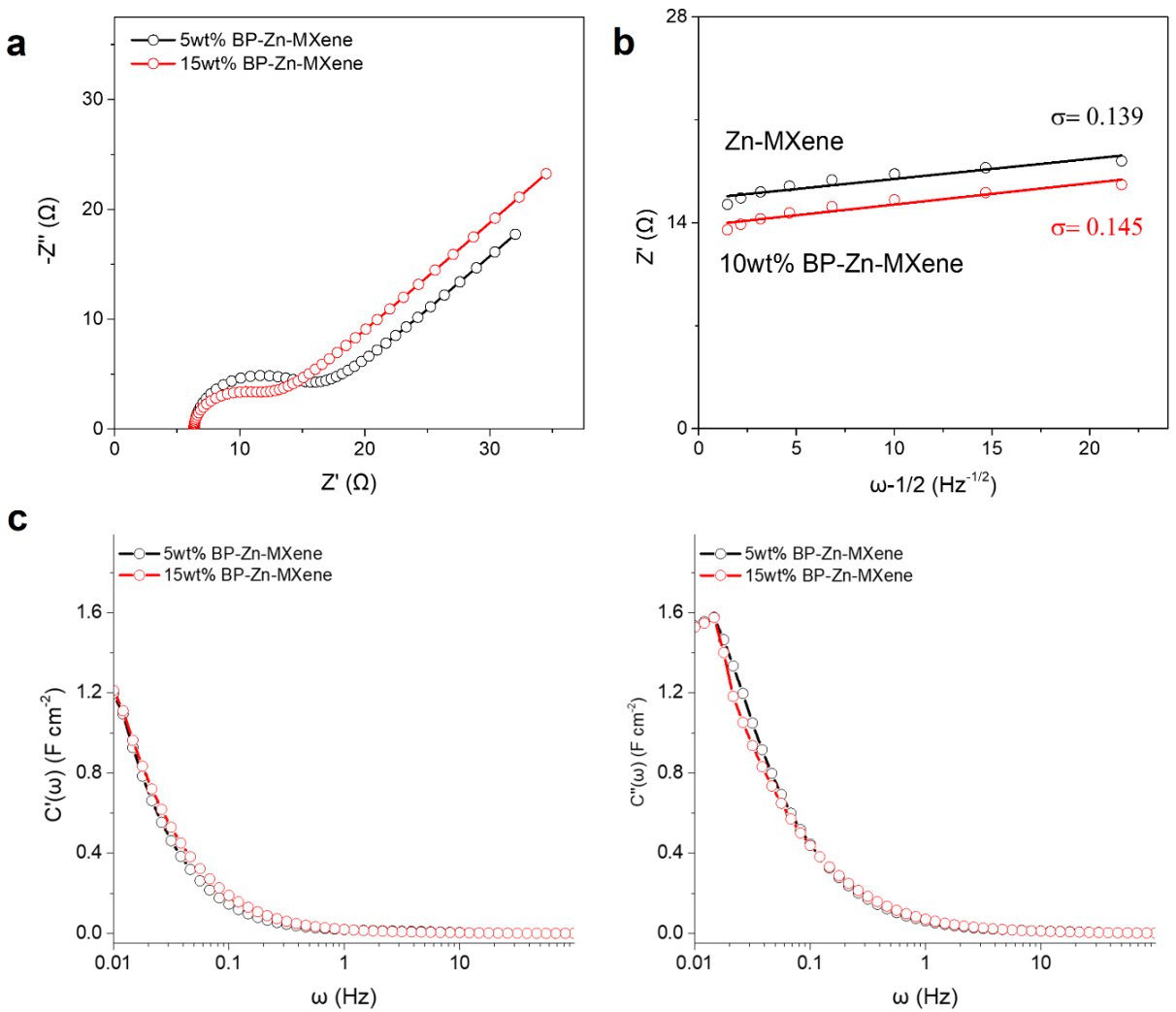


**Figure S12**. 5wt% BP-Zn-MXene and 15wt% BP-Zn-MXene electrodes in 2 M ZnSO_4_. a) Nyquist plots. b) Ion diffusion resistances. c) Real and imaginary capacitances.


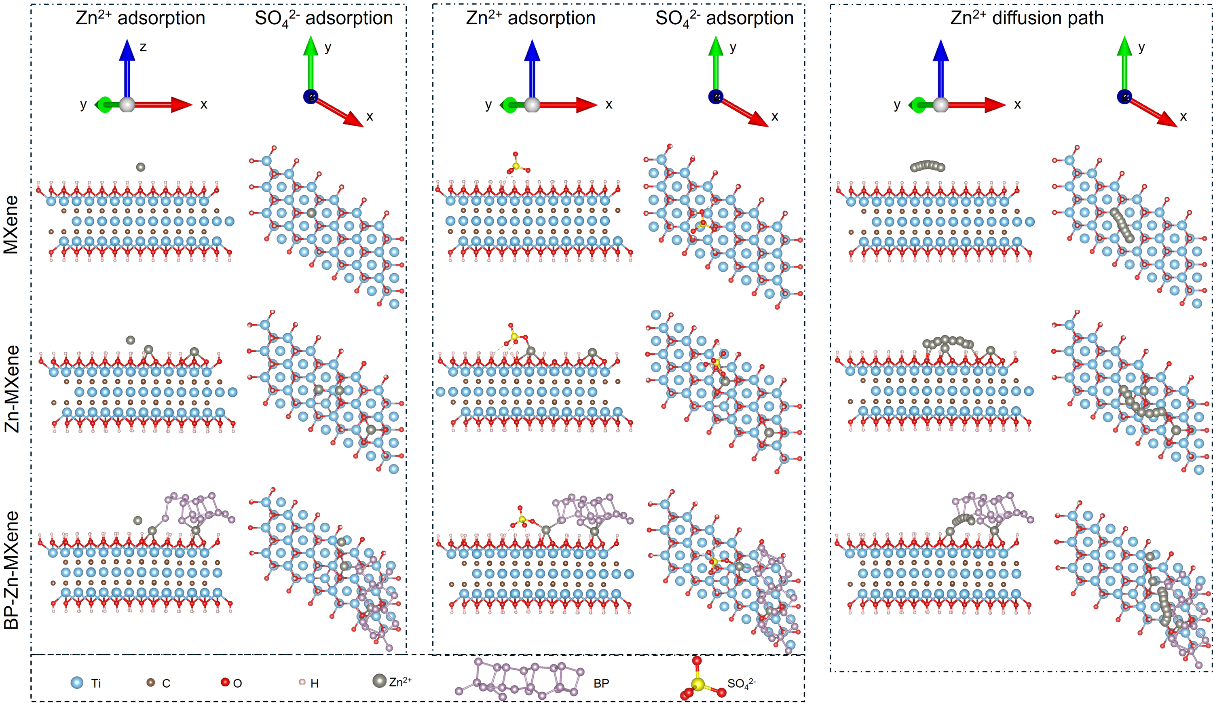


**Figure S13**. Different orientation views of the theoretical modes used to describe the Zn^2+^ adsorption, SO_4_^2-^ adsorption and Zn^2+^ diffusion path for MXene, Zn-MXene and BP-Zn-MXene structures.


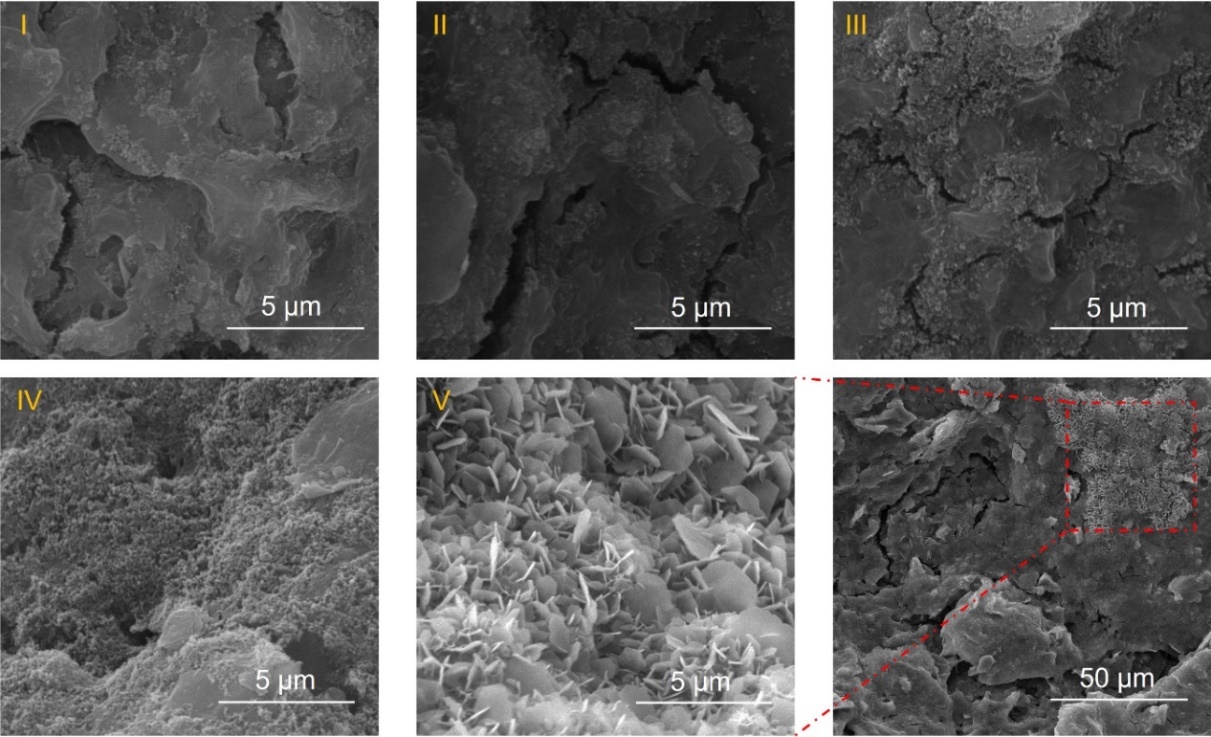


**Figure S14**. SEM images of 10wt% BP-Zn-MXene electrode corresponding to five various states during charge-discharge process at 1 mA cm^-2^.


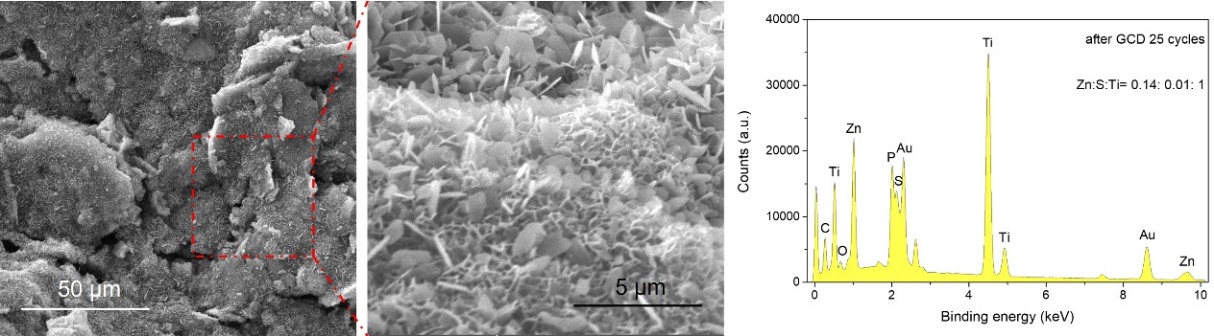


**Figure S15**. SEM images and corresponding EDS result of 10wt% BP-Zn-MXene electrode after 25 GCD cycles at 1 mA cm^-2^.


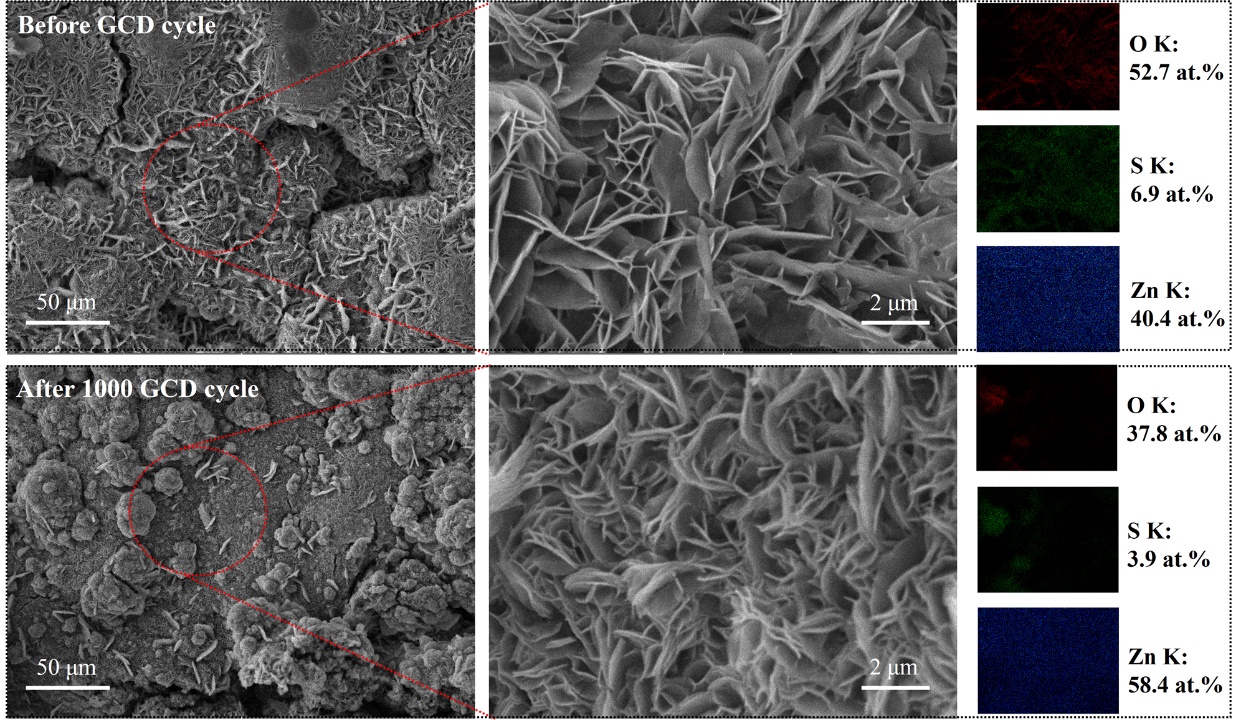


**Figure S16**. SEM images with EDS mapping results of the wearable ZIC electrodeposited Zn nanosheet anode before and after GCD1000 cycles at 5 mA.


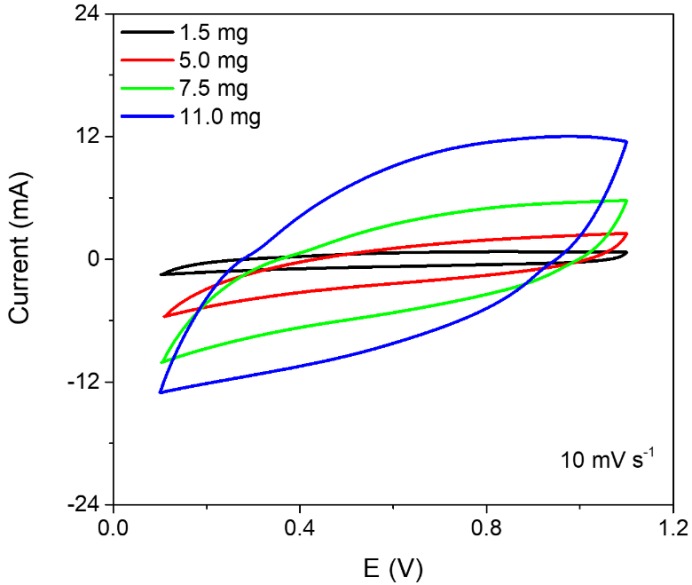


**Figure S17**. CV curves at 10 mV s^-1^ of wearable in-plane ZICs with different cathode load masses.


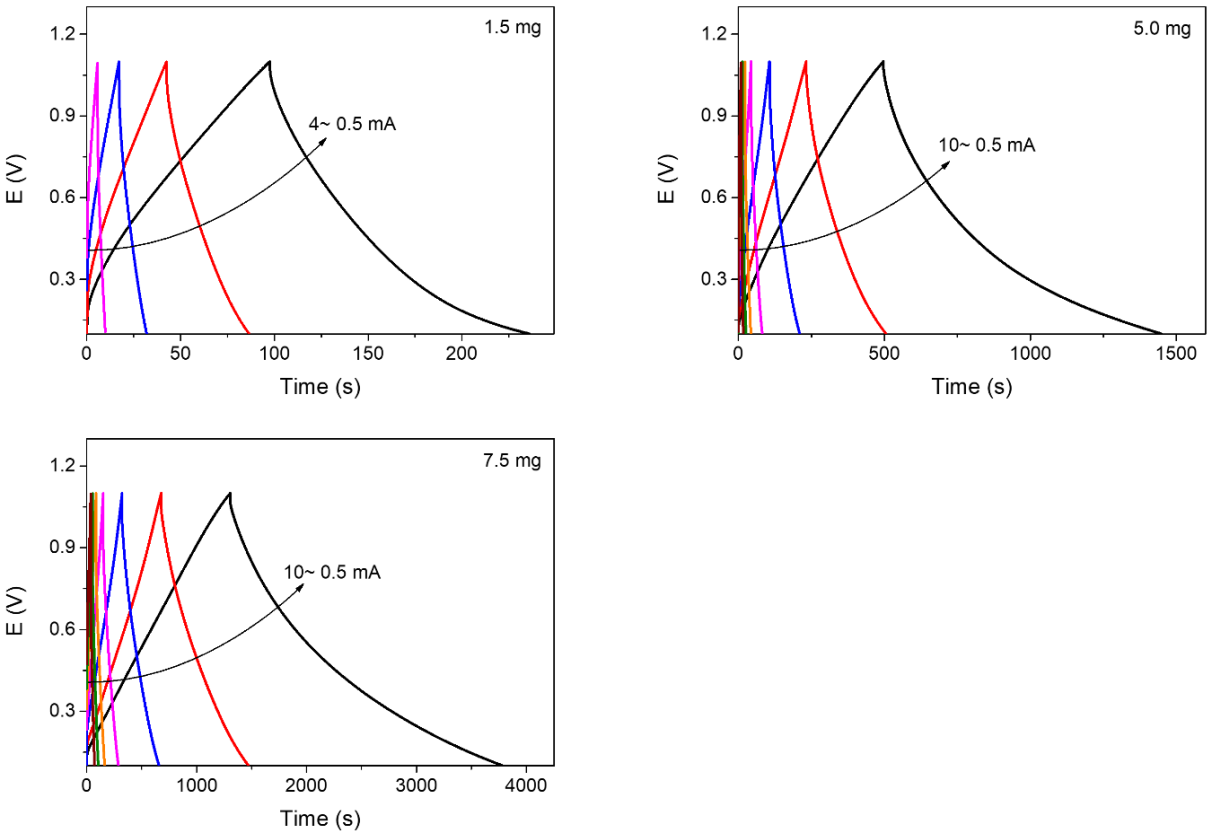


**Figure S18**. GCD profiles at current of 0.5~ 10 mA for wearable in-plane ZICs with various cathode load masses.


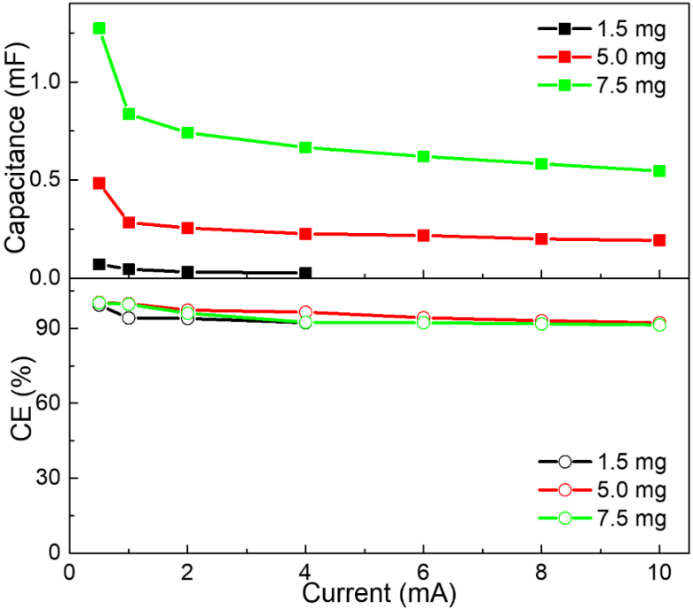


**Figure S19**. Capacities and CEs of wearable in-plane ZICs with various cathode load masses.


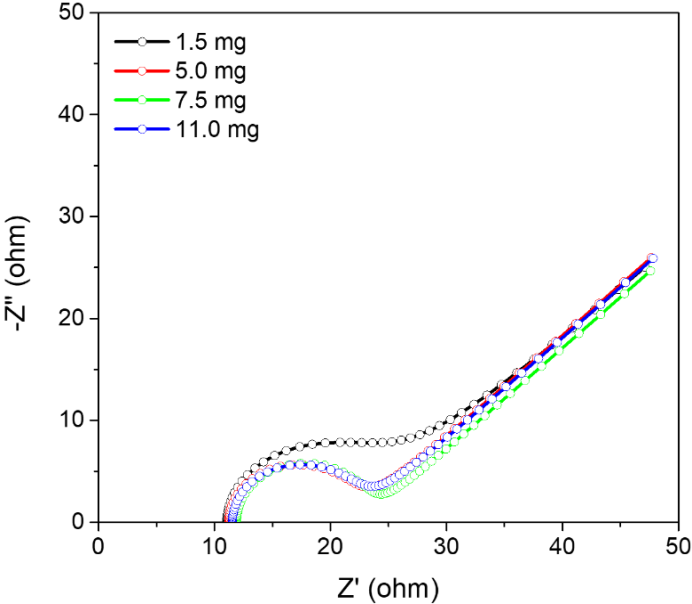


**Figure S****20** Nyquist plots of wearable in-plane ZICs with various cathode load masses.


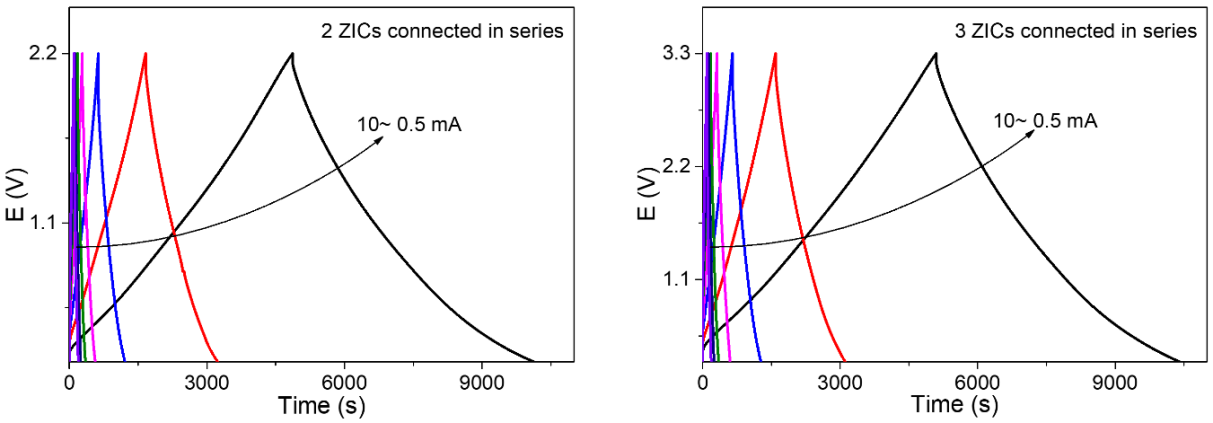


**Figure S21**. GCD curves of wearable in-plane ZICs connected in series.


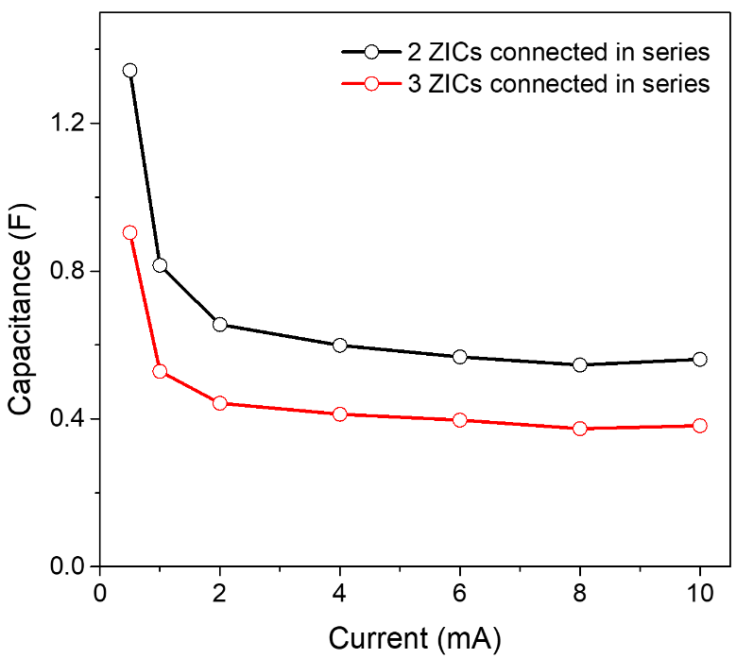


**Figure S22**. Capacities of wearable in-plane ZICs connected in series.


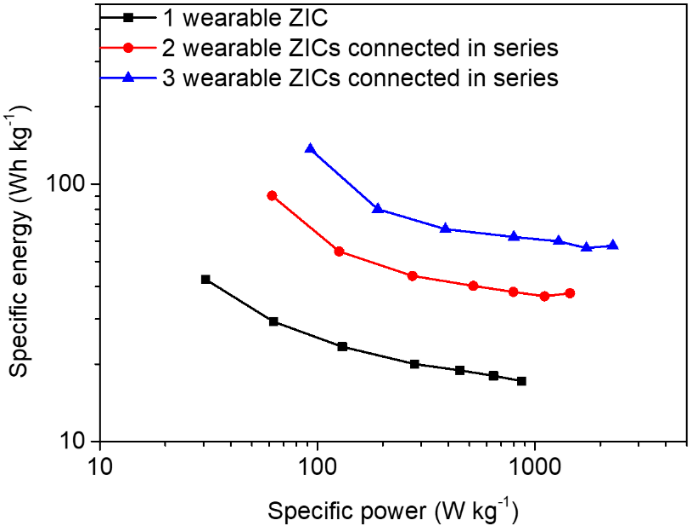


**Figure S23**. Ragone plot of wearable ZICs in terms of specific energy/power.


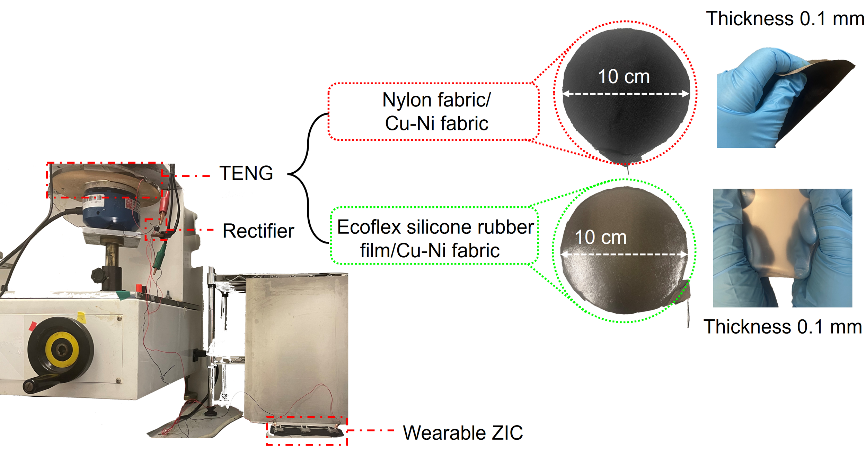


**Figure S24**. Illustration of integrated self-powered system by using fabric TENG and wearable in-plane ZIC device


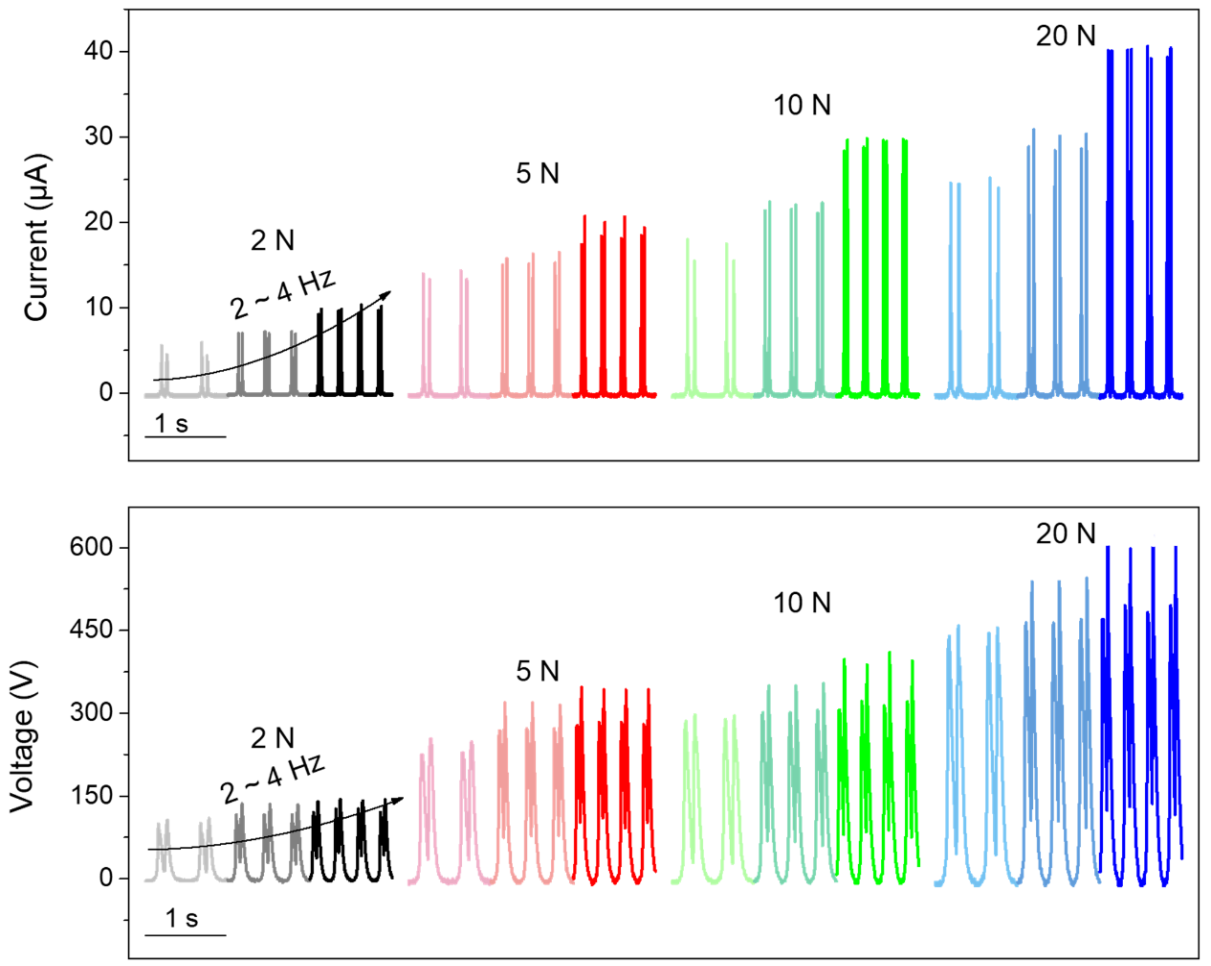


**Figure S25**. Output current and voltage signals of fabric TENG under various applied forces (2, 5, 10 and 20 N) and frequencies (2, 3 and 4 Hz).


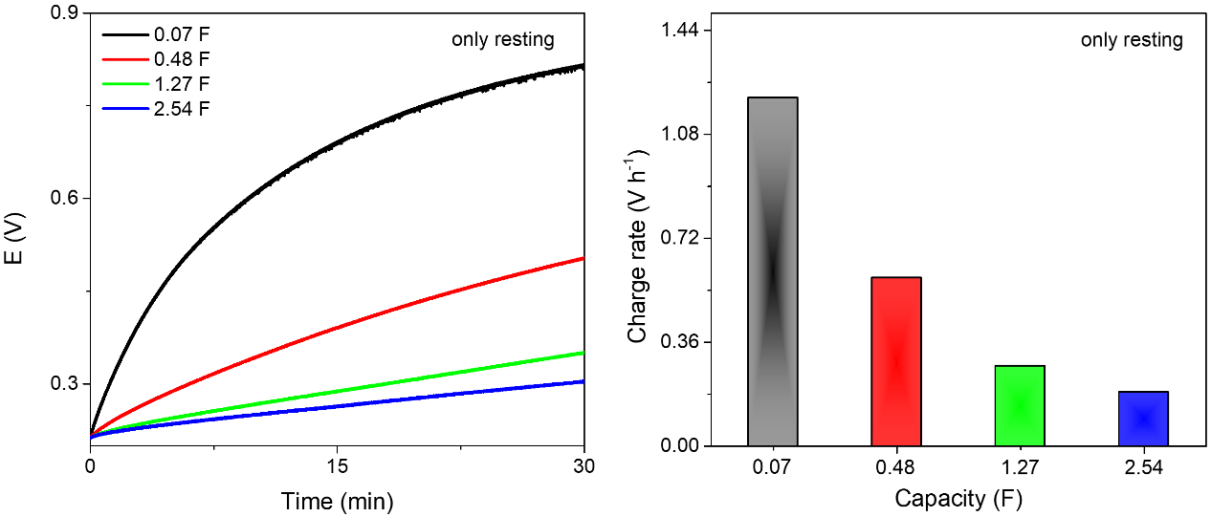


**Figure S26**. V_oc_ curves of self-powered device within 30 min of rest after being fully discharged.


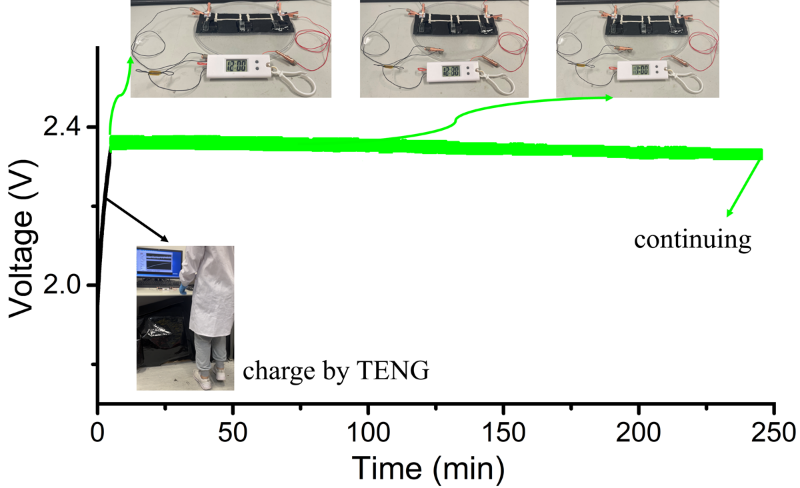


**Figure S27**. Illustration of charging-discharging process to power an electronic timer.

**Table S1** Recently reported self-discharge rates of ZIC and supercapacitor systems within 2 hours after fully charged.

| ZIC or supercapacitor system | Self-discharge rate  [mV h^-1^] | Refs. |
| --- | --- | --- |
| O/N doped fibrous carbon//Zn | ~150 | [11] |
| Sn^4+^-MXene//Zn | ~175 | [23] |
| BP//Zn | 100~ 200 | [30] |
| MXene//Zn | ~225 | [33] |
| TiN//Zn | ~200 | [34] |
| B/N doped carbon//Zn | ~125 | [35] |
| Active carbon//Zn | ~125 | [36] |
| porous carbon//Zn | 87.5 | [37] |
| CNT//CNT | 170 | [38] |
| MXene//MXene | ~200 | [39] |
| MXene//MXene | ~100 | [40] |
| PEDOT:PSS//PEDOT:PSS | ~100 | [41] |
| Manganese oxides//MXene | 86.2 | [42] |
| 10wt% BP-Zn-MXene//Zn | 46.5 | This work |

**Table S2** Recently reported electrochemical performance of quasi-solid-state MXene-based Zn-ion capacitors.

| MXene-based ZICs | Areal current  [mA cm^-2^] | Areal capacitance  [mF cm^-2^] | Areal energy  [μWh cm^-2^] | Refs. |
| --- | --- | --- | --- | --- |
| Ti_3_C_2_T_x_/PVA-Zn(OTf)_2_ | 0.75 | 1,432 | 389.7 | [18] |
| Ti_3_C_2_/Zn-CNT | 0.2 | ~500 | 100 | [19] |
| Ti_3_C_2_T_x_-Ag/PAM-ZnCl_2_ | 0.5 | ~1,153 | 117 | [20] |
| V_2_CT_x_/PAM-ZnSO_4_/Ti_3_C_2_T_x_ | 0.5 | 200.1 | 71.6 | [21] |
| MXene-Ag-BC/PAM-ZnCl_2_/Zn | 0.75 | 1,309 | 278.6 | [43] |
| MXene-Ag-BC/PAM-ZnCl_2_/Zn | 0.5 | 909 | 227 | [44] |
| MXene/PAM-Zn_S_O_4_/V_2_O_5_ | 0.34 | 129 | 48.9 | [45] |
| V_3_CrC_3_T_x_/PVA-ZnSO_4_/Zn | 0.2 | 278.3 | 51.1 | [46] |
| Ti_3_C_2_T_x_/PVA-ZnCl_2_/Zn | 10 mV s^-1^ | 72 | 20 | [47] |
| Ti_3_C_2_T_x_ PVA-ZnSO_4_/Zn | 0.5 | / | 249.9 | [48] |
| Wearable ZIC | 0.5 | 2,536.6 | 426.3 | This work |
